# Statistical Inference for Periodic Self-Exciting Threshold Integer-Valued Autoregressive Processes

**DOI:** 10.3390/e23060765

**Published:** 2021-06-17

**Authors:** Congmin Liu, Jianhua Cheng, Dehui Wang

**Affiliations:** 1 School of Mathematics, Jilin University, 2699 Qianjin Street, Changchun 130012, China; lcm18@mails.jlu.edu.cn (C.L.); chengjh@jlu.edu.cn (J.C.); 2 School of Economics, Liaoning University, Shenyang 110036, China

**Keywords:** periodic autoregression, integer-valued threshold models, parameter estimation

## Abstract

This paper considers the periodic self-exciting threshold integer-valued autoregressive processes under a weaker condition in which the second moment is finite instead of the innovation distribution being given. The basic statistical properties of the model are discussed, the quasi-likelihood inference of the parameters is investigated, and the asymptotic behaviors of the estimators are obtained. Threshold estimates based on quasi-likelihood and least squares methods are given. Simulation studies evidence that the quasi-likelihood methods perform well with realistic sample sizes and may be superior to least squares and maximum likelihood methods. The practical application of the processes is illustrated by a time series dataset concerning the monthly counts of claimants collecting short-term disability benefits from the Workers’ Compensation Board (WCB). In addition, the forecasting problem of this dataset is addressed.

## 1. Introduction

There has been considerable interest in integer-valued time series because of their wide range of applications, including epidemiology, finance, and disease modeling. Examples of such data are as follows: the number of major global earthquakes per year, monthly crimes in a particular country or region, and patient numbers in a hospital per month over a period of time, etc. Following the first-order integer-valued autoregressive (INAR(1)) models introduced by Al-Osh and Alzaid [1], INAR models have been widely used, see Du and Li [2], Jung et al. [3], Weiß [4], Ristić et al. [5], Zhang et al. [6], Li et al. [7], Kang et al. [8] and Yu et al. [9], among others. However, for so-called piecewise phenomenon such as high thresholds, sudden bursts of large values, and time volatility, the INAR model will not work well. The threshold models (Tong [10]; Tong and Lim [11]) have attracted much attention and have been widely used to model nonlinear phenomena. To capture the piecewise phenomenon of integer-valued time series, Monteiro et al. [12] introduced a class of self-exciting threshold integer-valued autoregressive (SETINAR) models driven by independent Poisson-distributed random variables. Wang et al. [13] proposed a self-excited threshold Poisson autoregressive (SETPAR) model. Yang et al. [14] considered a class of SETINAR processes that properly capture flexible asymmetric and nonlinear responses without assuming the distributions for the errors. Yang et al. [15] introduced an integer-valued threshold autoregressive process based on a negative binomial thinning operator (NBTINAR(1)).

In addition, there are many sources of business, economic and meteorology time series data showing a periodically varying phenomenon that repeats itself after a regular period of time. It may be affected by seasonal factors and human activities. For dealing with the processes exhibiting periodic patterns, Bennett [16] and Gladyshev [17] proposed periodically correlated random processes. Then, Bentarzi and Hallin [18], Lund and Basawa [19], Basawa and Lund [20], and Shao [21], among other authors, studied the periodic autoregressive moving-average (PARMA) models in some detail. To capture the periodic phenomenon of integer-valued time series, Monteiro et al. [22] proposed the periodic integer-valued autoregressive models of order one (PINAR(1)) with period *T*, driven by a periodic sequence of independent Poisson-distributed random variables. Hall et al. [23] considered the extremal behavior of periodic integer-valued moving-average sequences. Santos et al. [24] introduced a multivariate PINAR model with time-varying parameters. The analysis of periodic self-exciting threshold integer-valued autoregressive (PSETINAR${\left(2;1,1\right)}_{T}$) processes was introduced by Pereira et al. [25]. Manaa and Bentarzi [26] established the existence of high moment and the strict periodic stationarity for the PSETINAR${\left(2;1,1\right)}_{T}$ processes. The CLS and CML methods are applied to estimate the parameters while using the nested sub-sample search (NeSS) algorithm proposed by Li and Tong [27] to estimate the periodic threshold parameters. A drawback of this PSETINAR${\left(2;1,1\right)}_{T}$ model is that the mean and variance of Poisson distribution are equal, which is not always true in the real data. Therefore, in this paper, we remove the assumption of Poisson distribution, only specify the relationship between mean and variance of observations, develop quasi-likelihood inference for the PSETINAR${\left(2;1,1\right)}_{T}$ processes, and consider the estimation of thresholds.

Quasi-likelihood is a non-parametric inference method proposed by Wedderburn [28]. It is very useful in cases where the exact distributional information is not available, while only the relation between mean and variance of the observation is given, and it enjoys a certain robustness of validity. Quasi-likelihood has been widely applied. For example, Azrak and Mélard [29] proposed a simple and efficient algorithm to evaluate the exact quasi-likelihood of ARMA models with time-dependent coefficients; Christou and Fokianos [30] studied probabilistic properties and quasi-likelihood estimation for negative binomial time series models; Li et al. [31] studied the quasi-likelihood inference for the self-exciting threshold integer-valued autoregressive (SETINAR(2,1)) processes under a weaker condition; Yang et al. [32] modeled overdispersed or underdispersed count data with generalized Poisson integer-valued autoregressive (GPINAR(1)) processes and investigated the maximum quasi- likelihood estimators.

The remainder of this paper is organized as follows. In Section 2, we redefine the PSETINAR(2; 1, 1)${}_{T}$ processes under weak conditions and discuss their basic properties. In Section 3, we consider the quasi-likelihood inference for the unknown parameters. Thresholds estimation is also discussed. Section 4 presents some simulation results for the estimates. In Section 5, we give an application of the proposed processes to a real dataset. The forecasting problem of this dataset is addressed. Concluding remarks are given in Section 6. All proofs are postponed to the Appendix A.

## 2. The Model and Its Properties

The periodic self-exciting threshold integer-valued autoregressive model of order one with two regimes (PSETINAR${\left(2;1,1\right)}_{T}$) (originally proposed by Pereira et al. [25], and further studied by Manaa and Bentarzi [26]) is defined by the recursive equation:

(1) $$X_{t}=\left\{\begin{array}{cc} \alpha_{t}^{\left(1\right)}\circ X_{t-1}+Z_{t},\; & X_{t-1}\leq r_{t},\\ \alpha_{t}^{\left(2\right)}\circ X_{t-1}+Z_{t},\; & X_{t-1}>r_{t}, \end{array}\;t\in Z\right.$$

with threshold parameters $r_{t}=r_{j}$, autoregressive coefficients $\alpha_{t}^{\left(k\right)}=\alpha_{j}^{\left(k\right)}\in \left(0,1\right)$, for k=1,2, t=j+sT,j=1,2,⋯,T,s∈Z, and $T\in N_{0}$. Note that Equation (1) admits the representation

(2) $$X_{j+sT}=\left(\alpha_{j}^{\left(1\right)}\circ X_{j+sT-1}\right)I_{j+sT-1}^{\left(1\right)}+\left(\alpha_{j}^{\left(2\right)}\circ X_{j+sT-1}\right)I_{j+sT-1}^{\left(2\right)}+Z_{j+sT},$$

where

(i) $I_{j+sT-1}^{\left(1\right)}:=I\left\{X_{j+sT-1}\leq r_{j}\right\},I_{j+sT-1}^{\left(2\right)}:=1-I_{j+sT-1}^{\left(1\right)}=I\left\{X_{j+sT-1}>r_{j}\right\}$, in which $\left\{r_{j},j=1,2,\cdots ,T\right\}$ is a set of thresholds value;
(ii) The thinning operator “∘” is defined as
  (3) $$\alpha_{j}^{\left(k\right)}\circ X_{j+sT-1}=\sum_{i=1}^{X_{j+sT-1}}U_{i,j+sT}\left(\alpha_{j}^{\left(k\right)}\right),$$
  in which $\left\{U_{i,j+sT}\left(\alpha_{j}^{\left(k\right)}\right),j=1,2,\cdots ,T,s\in Z\right\}$ is a sequence of independent periodic Bernoulli random variables with $P\left(U_{i,j+sT}\left(\alpha_{j}^{\left(k\right)}\right)=1\right)=1-P\left(U_{i,j+sT}\left(\alpha_{j}^{\left(k\right)}\right)=0\right)=\alpha_{j}^{\left(k\right)},k=1,2$;
(iii) $\left\{Z_{j+sT},j=1,2,\cdots ,T,s\in Z\right\}$ constitutes a sequence of independent periodic random variables with $E\left(Z_{j+sT}\right)=\lambda_{j}$, $Var\left(Z_{j+sT}\right)=\sigma_{z,j}^{2}$, which is assumed to be independent of $\left\{X_{j+sT-1}\right\}$ and $\left\{\alpha_{j}^{\left(k\right)}\circ X_{j+sT-1}\right\}$.

**Remark** **1.** *The innovation of PSETINAR${\left(2;1,1\right)}_{T}$ process defined by Pereira et al. [25] and Manaa and Bentarzi [26] is a sequence of independent periodic Poisson-distributed random variables with mean $\lambda_{j}$, that is $\left\{Z_{t}\right\}∼P\left(\lambda_{j}\right)$, where t=j+sT, j=1,2,⋯,T, s∈Z. In this paper, we use $E\left(Z_{j+sT}\right)=\lambda_{j}$, $Var\left(Z_{j+sT}\right)=\sigma_{z,j}^{2}$ instead of the assumption of periodic Poisson distribution for $\left\{Z_{j+sT}\right\}$, so that the model is more flexible.*

The following proposition establishes the conditional mean and the conditional variance of the PSETINAR${\left(2;1,1\right)}_{T}$ process, which plays an important role in the study of the process properties and parameter estimations.

**Proposition** **1.** *For any fixed j=1,2,⋯,T, with $T\in N_{0}$, the conditional mean and the conditional variance of the process $\left\{X_{t}\right\}$ for t=j+sT and s∈Z defined in (2) are given by*

*(i)*  *$E\left(X_{j+sT}|X_{j+sT-1}\right)=\alpha_{j}^{\left(1\right)}X_{j+sT-1}I_{j+sT-1}^{\left(1\right)}+\alpha_{j}^{\left(2\right)}X_{j+sT-1}I_{j+sT-1}^{\left(2\right)}+\lambda_{j}$,*
*(ii)*  *$Var\left(X_{j+sT}|X_{j+sT-1}\right)=\sum_{k=1}^{2}\alpha_{j}^{\left(k\right)}\left(1-\alpha_{j}^{\left(k\right)}\right)X_{j+sT-1}I_{j+sT-1}^{\left(k\right)}+\sigma_{z,j}^{2}$.*

The following theorem states the ergodicity of the PSETINAR${\left(2;1,1\right)}_{T}$ process (2). This property is useful in deriving the asymptotic properties of the parameter estimators.

**Theorem** **1.** *For any fixed j=1,2,⋯,T, with $T\in N_{0}$, the process $\left\{X_{t}\right\}$ for t=j+sT and s∈Z defined in (2) is an ergodic Markov chain.*

## 3. Parameters Estimation

Suppose we have a series of observations $\left\{X_{j+sT},j=1,2,\cdots ,T,s\in N_{0}\right\}$ generated from the PSETINAR${\left(2;1,1\right)}_{T}$ process. The goal of this section is to estimate the unknown parameters vector $\beta =\left(\beta_{1},\cdots ,\beta_{3T}\right){}^{\prime }≜(\alpha_{1}^{\left(1\right)},\alpha_{1}^{\left(2\right)},\lambda_{1},\alpha_{2}^{\left(1\right)},\alpha_{2}^{\left(2\right)},\lambda_{2},\cdots ,\alpha_{T}^{\left(1\right)},$$\alpha_{T}^{\left(2\right)},\lambda_{T}){}^{\prime }$ and threshold parameters vector $r=\left(r_{1},r_{2},\cdots ,r_{T}\right){}^{\prime }$. This section is divided into two subsections. In Section 3.1, we estimate the parameters vector β by using the maximum quasi-likelihood (MQL) method when the thresholds value is known. We consider the maximum quasi-likelihood (MQL) and conditional least square (CLS) estimators of thresholds r in Section 3.2.

### 3.1. Estimation of Parameters β

As described in Proposition 1 (ii), we have the variance of $X_{t}$ conditional on $X_{t-1}$, let $\theta_{j}≜\left(\theta_{j}^{\left(1\right)},\theta_{j}^{\left(2\right)},\sigma_{z,j}^{2}\right){}^{\prime }$ with $\theta_{j}^{\left(k\right)}=\alpha_{j}^{\left(k\right)}\left(1-\alpha_{j}^{\left(k\right)}\right)$, k=1,2, j=1,2,⋯,T, then the $Var\left(X_{j+sT}|X_{j+sT-1}\right)$ admits the representation

$$V_{\theta_{j}}\left(X_{j+sT}|X_{j+sT-1}\right)\overset{△}{=}Var\left(X_{j+sT}|X_{j+sT-1}\right)=\theta_{j}^{\left(1\right)}X_{j+sT-1}I_{j+sT-1}^{\left(1\right)}+\theta_{j}^{\left(2\right)}X_{j+sT-1}I_{j+sT-1}^{\left(2\right)}+\sigma_{z,j}^{2},$$

for $\forall j=1,2,\cdots ,T,s\in N_{0}$.

As discussed in Wedderburn [28], we have the set of standard quasi-likelihood estimating equations:

(4) $$L\left(\beta \right)=\sum_{s=0}^{N-1}\sum_{j=1}^{T}\frac{X_{j+sT}-E\left(X_{j+sT}|X_{j+sT-1}\right)}{V_{\theta_{j}}\left(X_{j+sT}|X_{j+sT-1}\right)}\frac{\partial E\left(X_{j+sT}|X_{j+sT-1}\right)}{\partial \beta_{i}}=0,$$

for i=1,⋯,3T, where *N* is the total number of cycles. By solving (4), the quasi-likelihood estimator can be obtained.

This method is essentially a two-step estimation, if $\theta_{j}$ is unknown, we propose substituting a suitable consistent estimator of $\theta_{j}$ obtained by other means, getting modified quasi-likelihood estimating equations and then solving them for the primary parameters of interest. In the modified quasi- likelihood estimating equations, we replace $\theta_{j}$ with a suitable consistent estimator ${\hat{\theta }}_{j}$. For simplicity in notation, we define $V_{{\hat{\theta }}_{j}}^{-1}\overset{△}{=}V_{{\hat{\theta }}_{j}}^{-1}\left(X_{j+sT}|X_{j+sT-1}\right)$. This approach leads to the modified quasi-likelihood estimator ${\hat{\beta }}_{MQL}$ of β (see Zheng, Basawa and Datta [33]):

(5) $${\hat{\beta }}_{MQL}=Q_{N}^{-1}q_{N},$$

where

$$Q_{N}=\left[\begin{array}{cccc} Q_{1,N} & 0 & \cdots & 0\\ 0 & Q_{2,N} & \cdots & 0\\ \vdots & \vdots & \ddots & \vdots \\ 0 & 0 & \cdots & Q_{T,N} \end{array}\right],$$

and

$$q_{N}=\left(q_{1,N},q_{2,N},\cdots ,q_{T,N}\right){}^{\prime },$$

moreover, the 0’s are $\left(3\times 3\right)$-null matrices, $Q_{j,N}$ and $Q_{j,N}\;\left(j=1,2,\cdots ,T\right)$ given by

$$Q_{j,N}=\left[\begin{array}{ccc} \sum_{s=0}^{N-1}V_{{\hat{\theta }}_{j}}^{-1}X_{j+sT-1}^{2}I_{j+sT-1}^{\left(1\right)} & 0 & \sum_{s=0}^{N-1}V_{{\hat{\theta }}_{j}}^{-1}X_{j+sT-1}I_{j+sT-1}^{\left(1\right)}\\ 0 & \sum_{s=0}^{N-1}V_{{\hat{\theta }}_{j}}^{-1}X_{j+sT-1}^{2}I_{j+sT-1}^{\left(2\right)} & \sum_{s=0}^{N-1}V_{{\hat{\theta }}_{j}}^{-1}X_{j+sT-1}I_{j+sT-1}^{\left(2\right)}\\ \sum_{s=0}^{N-1}V_{{\hat{\theta }}_{j}}^{-1}X_{j+sT-1}I_{j+sT-1}^{\left(1\right)} & \sum_{s=0}^{N-1}V_{{\hat{\theta }}_{j}}^{-1}X_{j+sT-1}I_{j+sT-1}^{\left(2\right)} & \sum_{s=0}^{N-1}V_{{\hat{\theta }}_{j}}^{-1} \end{array}\right],$$

$$q_{j,N}=\left(\begin{array}{ccc} \sum_{s=0}^{N-1}V_{{\hat{\theta }}_{j}}^{-1}X_{j+sT}X_{j+sT-1}I_{j+sT-1}^{\left(1\right)} & \sum_{s=0}^{N-1}V_{{\hat{\theta }}_{j}}^{-1}X_{j+sT}X_{j+sT-1}I_{j+sT-1}^{\left(2\right)} & \sum_{s=0}^{N-1}V_{{\hat{\theta }}_{j}}^{-1}X_{j+sT} \end{array}\right){}^{\prime }.$$

Note that we use consistent estimator ${\hat{\theta }}_{j}=\left({\hat{\alpha }}_{j}^{\left(1\right)}\left(1-{\hat{\alpha }}_{j}^{\left(1\right)}\right),{\hat{\alpha }}_{j}^{\left(2\right)}\left(1-{\hat{\alpha }}_{j}^{\left(2\right)}\right),{\hat{\sigma }}_{z,j}^{2}\right){}^{\prime }$ instead of $\theta_{j}$.

Next, the proposition gives consistent estimators ${\hat{\sigma }}_{z,j}^{2}$ of $\sigma_{z,j}^{2}$, which depends on some consistent estimators ${\hat{\alpha }}_{j}^{\left(k\right)}$ and ${\hat{\lambda }}_{j}$ with k=1,2, j=1,2,⋯,T.

**Proposition** **2.** *The following variance estimators for $\left\{Z_{j+sT}\right\}$ with $j=1,2,\cdots ,T,s\in N_{0}$ are consistent:*

(6) $$\begin{array}{c} \left(i\right)\;{\hat{\sigma }}_{1,z,j}^{2}=\frac{1}{N}\sum_{s=0}^{N-1}{\left(X_{j+sT}-\sum_{k=1}^{2}{\hat{\alpha }}_{j}^{\left(k\right)}X_{j+sT-1}I_{j+sT-1}^{\left(k\right)}-{\hat{\lambda }}_{j}\right)}^{2}\\ \;\;\;\;\;-\frac{1}{N}\sum_{k=1}^{2}\sum_{s=0}^{N-1}{\hat{\alpha }}_{j}^{\left(k\right)}\left(1-{\hat{\alpha }}_{j}^{\left(k\right)}\right)X_{j+sT-1}I_{j+sT-1}^{\left(k\right)}, \end{array}$$

(7) $$\begin{array}{c} \left(ii\right)\;{\hat{\sigma }}_{2,z,j}^{2}={\hat{\sigma }}_{x,j}^{2}-{\hat{p}}_{j}\left[{\hat{\alpha }}_{j}^{{\left(1\right)}^{2}}{\hat{\sigma }}_{j}^{2^{\left(1\right)}}+{\hat{\alpha }}_{j}^{\left(1\right)}\left(1-{\hat{\alpha }}_{j}^{\left(1\right)}\right){\hat{\mu }}_{j}^{\left(1\right)}\right]\\ \;\;\;\;\;\;-\left(1-{\hat{p}}_{j}\right)\left[{\hat{\alpha }}_{j}^{{\left(2\right)}^{2}}{\hat{\sigma }}_{j}^{2^{\left(2\right)}}+{\hat{\alpha }}_{j}^{\left(2\right)}\left(1-{\hat{\alpha }}_{j}^{\left(2\right)}\right){\hat{\mu }}_{j}^{\left(2\right)}\right]\\ \;\;-{\hat{p}}_{j}\left(1-{\hat{p}}_{j}\right){\left({\hat{\alpha }}_{j}^{\left(1\right)}{\hat{\mu }}_{j}^{\left(1\right)}-{\hat{\alpha }}_{j}^{\left(2\right)}{\hat{\mu }}_{j}^{\left(2\right)}\right)}^{2}, \end{array}$$

*for $k=1,2,j=1,2,\cdots ,T,s\in N_{0}$, in which ${\hat{\alpha }}_{j}^{\left(k\right)}$ and ${\hat{\lambda }}_{j}$ are consistent estimators of $\alpha_{j}^{\left(k\right)}$ and $\lambda_{j}$ (for example, we can use the CLS estimators given in Theorem 3.1 of Pereira et al. [25]), furthermore*

$${\bar{X}}_{j}=\frac{1}{N}\sum_{s=0}^{N-1}X_{j+sT},\;{\hat{\sigma }}_{x,j}^{2}=\frac{1}{N}\sum_{s=0}^{N-1}{\left(X_{j+sT}-{\bar{X}}_{j}\right)}^{2},$$

$$N_{j}^{\left(k\right)}=\sum_{s=0}^{N-1}I_{j+sT-1}^{\left(k\right)},\;{\hat{\mu }}_{j}^{\left(k\right)}=\frac{1}{N_{j}^{\left(k\right)}}\sum_{s\in \{I_{j+sT-1}^{\left(k\right)}=1\}}X_{j+sT},$$

$$\;{\hat{p}}_{j}=\frac{1}{N}\sum_{s=0}^{N-1}I_{j+sT-1}^{\left(1\right)},\;{\hat{\sigma }}_{j}^{2^{\left(k\right)}}=\frac{1}{N_{j}^{\left(k\right)}}\sum_{s\in \{I_{j+sT-1}^{\left(k\right)}=1\}}{\left(X_{j+sT}-{\hat{\mu }}_{j}^{\left(k\right)}\right)}^{2}.$$

*The two estimations are based on conditional variance Var$\left(X_{j+sT}|X_{j+sT-1}\right)$ and variance Var$\left(X_{j+sT}\right)$, respectively. The details can be found in the Appendix A.*

To study the asymptotic behavior of the estimator ${\hat{\beta }}_{MQL}$, we make the following assumptions about the process of $\left\{X_{t}\right\}$:

(C1) *By Proposition 1 in Pereira et al. [25], we assume the $\left\{X_{t}\right\}$ is a strictly ciclostationary process;*
(C2) $E|X_{t}{|}^{4}<\infty$.

Now for the asymptotic properties of the quasi-likelihood estimator ${\hat{\beta }}_{MQL}$ given by (5), we have the following asymptotic distribution.

**Theorem** **2.** *Let $\left\{X_{t}\right\}$ be a PSETINAR${\left(2;1,1\right)}_{T}$ process defined in (2), then under the assumptions (C1)-(C2), the estimator ${\hat{\beta }}_{MQL}$ given by (5) is asymptotically normal,*

$$\sqrt{N}\left({\hat{\beta }}_{MQL}-\beta \right)\to N\left(0,H^{-1}\left(\theta \right)\right),$$

*where*

$$H\left(\theta \right)=\left[\begin{array}{cccc} H_{1}\left(\theta \right) & 0 & \cdots & 0\\ 0 & H_{2}\left(\theta \right) & \cdots & 0\\ \vdots & \vdots & \ddots & \vdots \\ 0 & 0 & \cdots & H_{T}\left(\theta \right) \end{array}\right],$$

*with matrices $H_{j}\;\left(j=1,2,\cdots ,T\right)$ given by*

$$H_{j}\left(\theta \right)=\left[\begin{array}{ccc} E\left(V_{\theta_{j}}^{-1}\left(X_{j}|X_{j-1}\right)X_{j-1}^{2}I_{j-1}^{\left(1\right)}\right) & 0 & E\left(V_{\theta_{j}}^{-1}\left(X_{j}|X_{j-1}\right)X_{j-1}I_{j-1}^{\left(1\right)}\right)\\ 0 & E\left(V_{\theta_{j}}^{-1}\left(X_{j}|X_{j-1}\right)X_{j-1}^{2}I_{j-1}^{\left(2\right)}\right) & E\left(V_{\theta_{j}}^{-1}\left(X_{j}|X_{j-1}\right)X_{j-1}I_{j-1}^{\left(2\right)}\right)\\ E\left(V_{\theta_{j}}^{-1}\left(X_{j}|X_{j-1}\right)X_{j-1}I_{j-1}^{\left(1\right)}\right) & E\left(V_{\theta_{j}}^{-1}\left(X_{j}|X_{j-1}\right)X_{j-1}I_{j-1}^{\left(2\right)}\right) & E\left(V_{\theta_{j}}^{-1}\left(X_{j}|X_{j-1}\right)\right) \end{array}\right].$$

It is worth mentioning that this theorem reflects the consistency of the estimator ${\hat{\beta }}_{MQL}$.

### 3.2. Estimation of Thresholds Vector r

Note that in the real data application, the threshold values are also unknown. In this subsection, we estimate the thresholds vector $r={\left(r_{1},r_{2},\cdots ,r_{T}\right)}^{\prime }$. Here, we further promote the nested sub-sample search (NeSS) algorithm (see, e.g., Yang et al. [15], Li and Tong [27], and Li et al. [31]) and use conditional least squares (CLS) and modified quasi-likelihood (MQL) principles to estimate r.

For some fixed $\lambda ={\left(\lambda_{1},\lambda_{2},\cdots ,\lambda_{T}\right)}^{\prime }$, the application of the conditional least squares principle yields the sum of squared errors:

$$\begin{array}{cc} \; & S_{N}\left(r,\lambda \right)\\ = & \sum_{s=0}^{N-1}\sum_{j=1}^{T}{\left(X_{j+sT}-\sum_{k=1}^{2}\frac{\sum_{s=0}^{N-1}\left(X_{j+sT}X_{j+sT-1}I_{j+sT-1}^{\left(k\right)}-\lambda_{j}X_{j+sT-1}I_{j+sT-1}^{\left(k\right)}\right)}{\sum_{s=0}^{N-1}X_{j+sT-1}^{2}I_{j+sT-1}^{\left(k\right)}}X_{j+sT-1}I_{j+sT-1}^{\left(k\right)}-\lambda_{j}\right)}^{2}, \end{array}$$

and then the thresholds vector r can be estimated by minimizing $S_{N}\left(r,\lambda \right)$,

(8) $$\hat{r}=\arg\min_{r\in \left[\underline{r},\bar{r}\right]}S_{N}\left(r,\lambda \right),$$

where $\underline{r}$ and $\bar{r}$ are some known lower and upper bounds of r. In practice, they can be selected as the minimum and maximum values in each cycle of the sample. For convenience, we consider an alternative objective function

$$J_{N}\left(r,\lambda \right)=S_{N}-S_{N}\left(r,\lambda \right),$$

where

$$S_{N}=\sum_{s=0}^{N-1}\sum_{j=1}^{T}{\left(X_{j+sT}-\frac{\sum_{s=0}^{N-1}\left(X_{j+sT}X_{j+sT-1}-\lambda_{j}X_{j+sT-1}\right)}{\sum_{s=0}^{N-1}X_{j+sT-1}^{2}}X_{j+sT-1}-\lambda_{j}\right)}^{2}.$$

Now, the optimization in (8) is equivalent to

(9) $${\hat{r}}_{CLS}=\arg\max_{r\in \left[\underline{r},\bar{r}\right]}J_{N}\left(r,\lambda \right),$$

where ${\hat{r}}_{CLS}$ is the conditional least squares estimator of the thresholds vector r.

Inspired by the method of conditional least squares, we investigate the performances of r by using the quasi-likelihood principle. The modified quasi-likelihood estimator ${\hat{r}}_{MQL}$ of r is obtained by maximizing the expression

$${\tilde{J}}_{N}\left(r,\lambda \right)={\tilde{S}}_{N}-{\tilde{S}}_{N}\left(r,\lambda \right),$$

which yields

(10) $${\hat{r}}_{MQL}=\arg\max_{r\in \left[\underline{r},\bar{r}\right]}{\tilde{J}}_{N}\left(r,\lambda \right),$$

where

$$\begin{array}{cc} \; & {\tilde{S}}_{N}\left(r,\lambda \right)\\ = & \sum_{s=0}^{N-1}\sum_{j=1}^{T}V_{{\hat{\theta }}_{j}}^{-1}{\left(X_{j+sT}\;-\;\sum_{k=1}^{2}\frac{\sum_{s=0}^{N-1}V_{{\hat{\theta }}_{j}}^{-1}\cdot \left(X_{j+sT}X_{j+sT-1}I_{j+sT-1}^{\left(k\right)}\;-\;\lambda_{j}X_{j+sT-1}I_{j+sT-1}^{\left(k\right)}\right)}{\sum_{s=0}^{N-1}V_{{\hat{\theta }}_{j}}^{-1}\cdot X_{j+sT-1}^{2}I_{j+sT-1}^{\left(k\right)}}X_{j+sT-1}I_{j+sT-1}^{\left(k\right)}\;-\;\lambda_{j}\right)}^{2}, \end{array}$$

and

$${\tilde{S}}_{N}=\sum_{s=0}^{N-1}\sum_{j=1}^{T}V_{{\hat{\theta }}_{j}}^{-1}{\left(X_{j+sT}-\frac{\sum_{s=0}^{N-1}V_{{\hat{\theta }}_{j}}^{-1}\cdot \left(X_{j+sT}X_{j+sT-1}-\lambda_{j}X_{j+sT-1}\right)}{\sum_{s=0}^{N-1}V_{{\hat{\theta }}_{j}}^{-1}\cdot X_{j+sT-1}^{2}}X_{j+sT-1}-\lambda_{j}\right)}^{2}.$$

**Figure 1:**
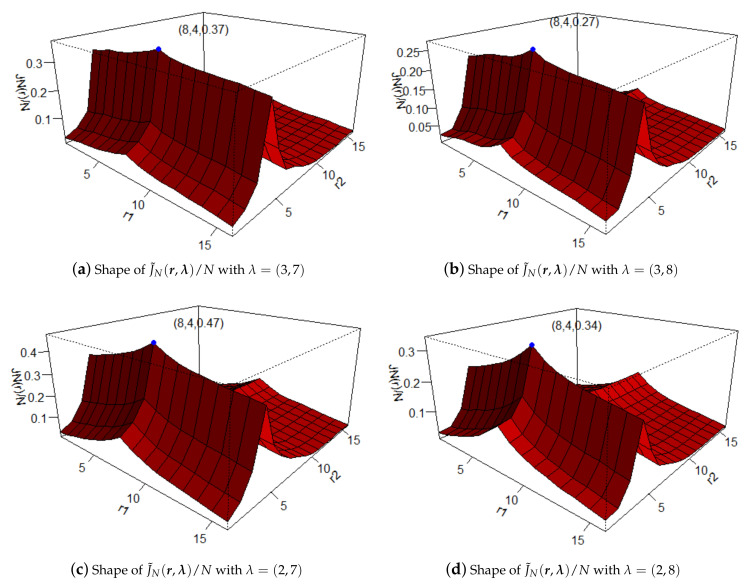
The shapes of ${\tilde{J}}_{N}\left(r,\lambda \right)/N$.

It is worth mentioning that there are unknown parameters $\lambda_{j}$ with j=1,⋯,T when we use (9) and (10) to estimate thresholds vector r. As argued in Li and Tong [27], Yang et al. [14], and Yang et al. [15], when λ and *r* are one-dimensional parameters, we can choose any positive number as the value of λ without worrying about getting a wrong result of $\hat{r}$. Fortunately, we also find out by simulations that the estimations of r by maximizing ${\tilde{J}}_{N}\left(r,\lambda \right)$ and $J_{N}\left(r,\lambda \right)$ do not depend on the value of λ. In order to give an intuitive impression of ${\tilde{J}}_{N}\left(r,\lambda \right)/N$, we generate a set of data with Model I (given in Section 4, i.e., $T=2,N=50,\beta ={\left(0.2,0.1,3,0.8,0.1,7\right)}^{\prime },r={\left(8,4\right)}^{\prime }$), and plot the shapes of ${\tilde{J}}_{N}\left(r,\lambda \right)/N$. From Figure 1, we can see that for different values of λ, the shape of ${\tilde{J}}_{N}\left(r,\lambda \right)/N$ changes, but the maximum value in each subfigure is obtained at the true thresholds vector $r={\left(8,4\right)}^{\prime }$. In practice, we can choose the mean in each cycle of the samples for $\lambda_{j},j=1,2,\cdots ,T$.

Actually, using the quasi-likelihood method to estimate the thresholds is a three-step estimation procedure, and we now present the algorithm to implement our estimation procedure as follows:

Step 1: *Choose the upper bound $\bar{r}$ and lower bound $\underline{r}$ of r, solve (9) to get the ${\hat{r}}_{CLS}$ with $\lambda_{j}={\bar{X}}_{j}=\frac{1}{N}\sum_{s=0}^{N-1}X_{j+sT},j=1,2,\cdots ,T$;*
Step 2: *Fix ${\hat{r}}_{CLS}$ at the current value, solve (6) or (7) to get the ${\hat{\sigma }}_{z,j}^{2},j=1,2,\cdots ,T$, where $\alpha_{j}^{\left(k\right)}$ and $\lambda_{j}$ with k=1,2 can be estimated by other methods, then solve (5) to get ${\hat{\beta }}_{MQL}$.*
Step 3: *Fix ${\hat{\theta }}_{j}=\left({\hat{\alpha }}_{j,MQL}^{\left(1\right)}\left(1-{\hat{\alpha }}_{j,MQL}^{\left(1\right)}\right),{\hat{\alpha }}_{j,MQL}^{\left(2\right)}\left(1-{\hat{\alpha }}_{j,MQL}^{\left(2\right)}\right),{\hat{\sigma }}_{z,j}^{2}\right){}^{\prime },j=1,2,\cdots ,T$ at its estimated value from Step 2, choose the same upper bound $\bar{r}$ and lower bound $\underline{r}$ as in Step 1, solve (10) to get ${\hat{r}}_{MQL}$.*

## 4. Simulation Study

**Figure 2:**
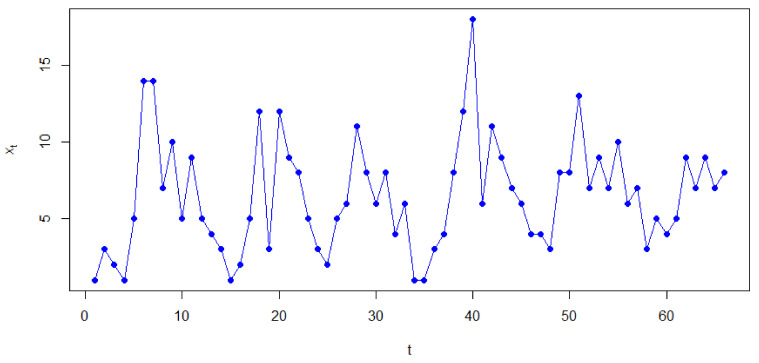
Sample path of the first six cycles.

In this section, we conduct simulation studies to illustrate the finite sample performances of the estimates. The initial value $X_{0}$ is fixed at 0. In order to capture the characteristics of the data from the PSETINAR${\left(2;1,1\right)}_{T}$ process, we first generate a set of data with the distribution of innovations $\left\{Z_{t}\right\}$ given by Model I (mentioned below in this section) and parameters β=(0.2,0.45,1,0.2,0.45,2,0.65,0.45, 1, 0.65,0.45,2,0.2,0.45,3,0.2,0.45, 7,0.8,0.45,7,0.2,0.1,3, 0.8, ${0.1,7,0.2,0.1,7,0.8,0.45,2)}^{\prime }$, r = ${\left(3,3,3,1,3,3,5,9,3,6,7\right)}^{\prime }$, T=11, N=50. The parameter vectors we choose here are randomly selected, and there are slight differences between the parameters of each cycle, the thresholds vector of r was chosen such that there are enough data in each regime. We give the sample path in the first six cycles in Figure 2, of which N=6. We can see that even if there are slight differences between the parameters of each cycle, the dataset still exhibits periodic characteristics.

To report the performances of the estimates, we conduct simulation studies under the following three models:

Model I. Assume that $\left\{Z_{t}\right\}$ is a sequence of i.i.d periodic Poisson distributed random variables with mean $E\left(Z_{t}\right)=Var\left(Z_{t}\right)=\lambda_{j}$ for $t=j+sT,j=1,2,\cdots ,T,s\in N_{0}$.

Model II. Assume that $\left\{Z_{t}\right\}$ is a sequence of i.i.d. periodic Geometric distributed random variables with p.m.f. given by

$$p\left(Z_{j+sT}=z\right)=\frac{\lambda_{j}^{z}}{{\left(1+\lambda_{j}\right)}^{1+z}},z=0,1,2,\cdots$$

with $E\left(Z_{t}\right)=\lambda_{j},Var\left(Z_{t}\right)=\lambda_{j}\left(1+\lambda_{j}\right)$ for $t=j+sT,j=1,2,\cdots ,T,s\in N_{0}$.

Model III. Assume that $\left\{Z_{t}\right\}$ is a sequence of i.i.d mixed distributed random variables,

$$Z_{t}=\Delta_{t}Z_{1t}+\left(1-\Delta_{t}\right)Z_{2t},$$

where $\left\{\Delta_{t}\right\}$ is a sequence of i.i.d periodic Bernoulli distributed random variables with $P\left(\Delta_{t}=1\right)=1-P\left(\Delta_{t}=0\right)=\rho_{j},\rho =\left(\rho_{1},\rho_{2},\cdots ,\rho_{T}\right)$ for $t=j+sT,j=1,2,\cdots ,T,s\in N_{0}$, which is independent of $\{Z_{it}\},i=1,2$.

For $\left\{Z_{1t}\right\}$ given in Model I and $\left\{Z_{2t}\right\}$ given in Model II, we can easily see that $E\left(Z_{t}\right)=\lambda_{j},Var\left(Z_{t}\right)=\lambda_{j}^{2}\left(1-\rho_{j}\right)+\lambda_{j}$.

For each model, we generate the data with $X_{0}=0$, set T=3 and the sample sizes n=NT=150,300,900. All the calculations are performed under the R3.6.2 software with 1000 replications. We use the command *constrOptim* to optimize the objective function of the maximum likelihood estimation. The threshold vector is calculated by the algorithms discussed in Section 3.2. Other algorithms are based on the explicit expressions.

### 4.1. Performances of the ${\hat{\beta }}_{\mathrm{CLS}}$, ${\hat{\beta }}_{\mathrm{MQL}}$ and ${\hat{\beta }}_{\mathrm{CML}}$

Pereira et al. [25] provided a theoretical basis for the conditional least squares (CLS) and conditional maximum likelihood (CML) estimators of the parameters vector β in the PSETINAR${\left(2;1,1\right)}_{T}$ process but did not conduct simulation research. Manaa and Bentarzi [26] provided the asymptotic properties of the estimators and compared their performance through a simulation study. To compare the performance of the three estimators ${\hat{\beta }}_{CLS}$, ${\hat{\beta }}_{CML}$ and ${\hat{\beta }}_{MQL}$ (given in Section 3), we conduct simulation studies for these three estimators under Models I to III. The parameters are selected as follows:

Series A. $\beta ={\left(0.2,0.45,1,0.2,0.45,2,0.8,0.45,2\right)}^{\prime },r={\left(3,2,2\right)}^{\prime }$.

Series B. $\beta ={\left(0.65,0.45,1,0.65,0.45,2,0.35,0.45,2\right)}^{\prime },r={\left(2,2,3\right)}^{\prime }$.

Series C. $\beta ={\left(0.2,0.45,3,0.2,0.45,7,0.8,0.45,7\right)}^{\prime },r={\left(12,7,9\right)}^{\prime }$.

To eliminate the influence of the change of parameters on estimates, we choose the series randomly and change the parameters with fixed $\alpha^{\left(k\right)},k=1,2$ or λ separately. The selection of these thresholds ensures there are enough data in each regime.

Spectral analysis starts from finding hidden periodicity, and it is an important subject of time series frequency domain analysis. The approach for studying hidden periods based on frequency domain analysis is the periodogram method, proposed by Schuster [34]; the rigorous examination is shown in Fisher [35]. For a series of observations $\{X_{t}\},t=1,2,\cdots ,n$, the periodogram is defined as

(11) $$I_{n}\left(f_{k}\right)=\frac{1}{n}{\left|\sum_{t=1}^{n}X_{t}e^{-i2\pi f_{k}t}\right|}^{2}=a_{k}^{2}+b_{k}^{2},$$

where

$$a_{k}=\left\{\begin{array}{cc} \frac{1}{\sqrt{n}}{\left(\sum_{t=1}^{n}X_{t}\cos\left(2\pi f_{k}t\right)\right)}^{2}, & k=1,2,\cdots ,\left[\frac{n-1}{2}\right],\\ \frac{1}{\sqrt{n}}\sum_{t=1}^{n}{\left(-1\right)}^{t}X_{t}, & k=\frac{n}{2}, \end{array}\right.$$

$$b_{k}=\left\{\begin{array}{cc} \frac{1}{\sqrt{n}}{\left(\sum_{t=1}^{n}X_{t}\sin\left(2\pi f_{k}t\right)\right)}^{2}, & k=1,2,\cdots ,\left[\frac{n-1}{2}\right],\\ 0, & k=\frac{n}{2}, \end{array}\right.$$

and the period $T=[1/\arg\max_{f}I_{n}\left(f_{k}\right)]$, where $\left[\cdot \right]$ denotes the integer part of a number.

**Figure 3:**
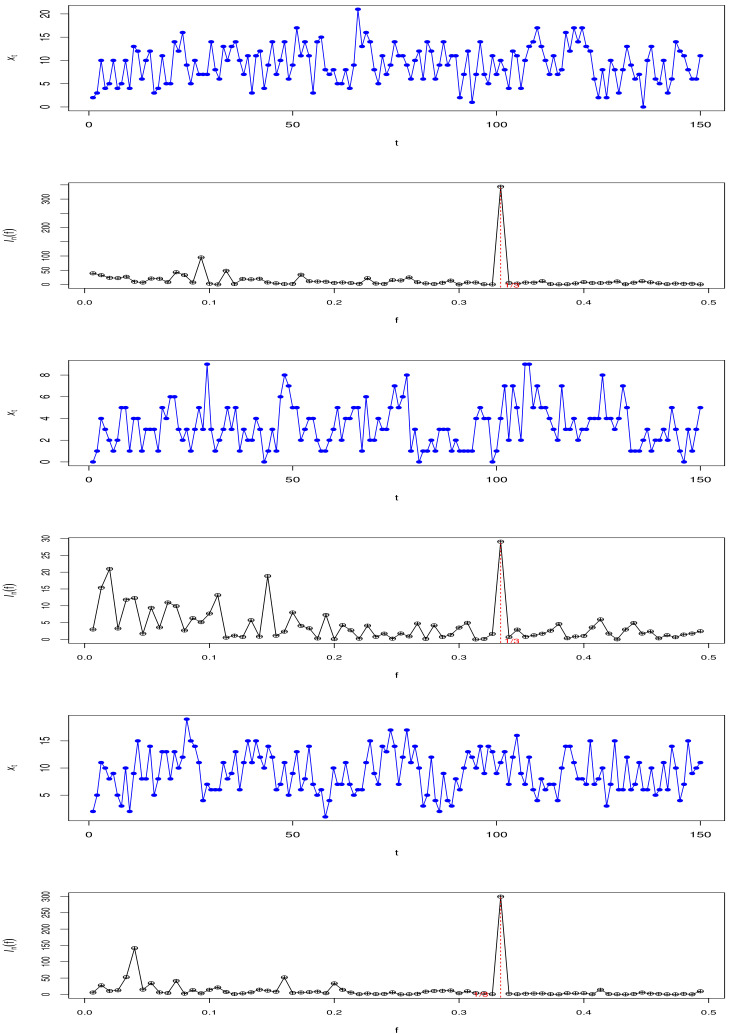
The sample path and periodogram of Series A(top), B(middle) and C(bottom) in Model I.

The sample path and periodogram of the Series A, B and C under Model I are plotted in Figure 3 to show the periodic characteristics. Because the period is three and short, it is difficult to see the period from the sample path, but the periodogram can show the period very well. In addition, the simulation results are summarized in Table 1, Table 2, Table 3, Table 4, Table 5, Table 6, Table 7, Table 8 and Table 9.

As expected, biases and MSE of the estimators decrease as the sample size *N* increases, which is in agreement with the asymptotic properties of the estimators: asymptotic unbiasedness and consistency. Most of the biases and MSE in Model II are larger than those in Model I. Maybe this is because the variance of $\left\{Z_{t}\right\}$ in Model II is larger than that in Model I, which leads to the fluctuation of data.

Table 1, Table 2, Table 3, Table 4, Table 5 and Table 6 summarize the simulation results for different series under Model I and Model II. From these tables, we can see that most of the biases and MSE of ${\hat{\beta }}_{MQL}$ are smaller than ${\hat{\beta }}_{CLS}$. Perhaps it is because that the MQL method uses more information about the data than the CLS method. Therefore, the MQL method can obtain the optimal value more accurately. In addition, most of the biases of ${\hat{\beta }}_{MQL}$ are smaller than ${\hat{\beta }}_{CML}$, while the MSE is larger, which is because the CML uses the distribution. If the distribution is correct, it is indeed better than the MQL. It is worth mentioning that the CML method is more complicated and time-consuming than the MQL method in the simulation procedure. We can conclude that the MQL estimators are better than CLS estimators, and the CML estimators are not unanimously better than MQL estimators.

To demonstrate the robustness of the MQL method, we consider the simulations about Model III with different series by using CLS, MQL and CML methods, and set N=300, $\rho =\left(0.9,0.9,0.9\right),\left(0.8,0.8,0.8\right)$, respectively. From Table 7, Table 8 and Table 9, we can see that when ρ varies from (0.9, 0.9, 0.9) down to (0.8, 0.8, 0.8), the effect on CLS and MQL estimators is slight. Most of the biases and MSE of MQL estimators are smaller than CLS. But due to incorrect distribution used, the biases and MSE of CML estimators increase. This indicates that the MQL method is more robust than CLS and CML methods.

### 4.2. Performances of ${\hat{r}}_{\mathrm{MQL}}$ and ${\hat{r}}_{\mathrm{CLS}}$

As discussed in Section 3.2, we estimate the thresholds vector by using conditional least squares and modified quasi-likelihood methods. The performances of ${\hat{r}}_{MQL}$ and ${\hat{r}}_{CLS}$ are compared in this subsection through simulation studies. From the simulation results in Section 4.1, we find that the contaminated data generated from Model III has little influence on least squares and quasi-likelihood estimators, so we only simulate thresholds estimation for different series under Model I and Model II. We assess the performance of r by the bias, MSE and bias median, where the bias median is defined by:

$$\mathrm{Bias}\;\mathrm{median}=\underset{i\in \{1,2,\cdots ,K\}}{\mathrm{median}}\left({\hat{r}}_{ij}-r_{0j}\right),j=1,2,\cdots ,T,$$

where ${\hat{r}}_{ij}$ is the estimator of $r_{0j}$, $r_{0j}$ is the true value with j=1,2,⋯,T, and *K* is the number of replications. The simulation results are summarized in Table 10, Table 11, Table 12, Table 13, Table 14 and Table 15.

From Table 10, Table 11, Table 12, Table 13, Table 14 and Table 15, we can see that all the simulation results perform better as sample size *N* increases, which implies that the estimators are consistent. The results in Table 10, Table 11 and Table 12 have smaller biases, bias medians and MSE than in Table 13, Table 14 and Table 15. This might be because the variance of Model II is larger than Model I for each series. Moreover, almost all the biases, bias medians and MSE of MQL estimators are smaller than CLS estimators, and the MSE of some MQL estimators are even half of the CLS. Because the thresholds are integer values, when we assess the accuracy of the estimators, the bias medians estimated can be more reasonable. It is concluded that it is much better to estimate the thresholds with the MQL method than CLS.

In the process of simulation, we generate the data with $X_{0}=0$; however, 0 is not the mean of the process, so we generate a set of data, discard some data generated first, and use the remaining data for inference, namely, “burn in” samples. Here, we generate a set of data with a length of 1800. We do the simulations for Series A of Model I, Model II and Model III $\left(\rho =\left(0.8,0.8,0.8\right)\right)$. Other simulation settings are the same as before. The simulation results are listed in Table 16, Table 17, Table 18, Table 19 and Table 20. From these tables, we can see that under the “burn in” samples, the estimated results are similar to that when the initial value is 0, which indicates that the initial value will not affect our estimated results.

## 5. Real Data Example

**Figure 4:**
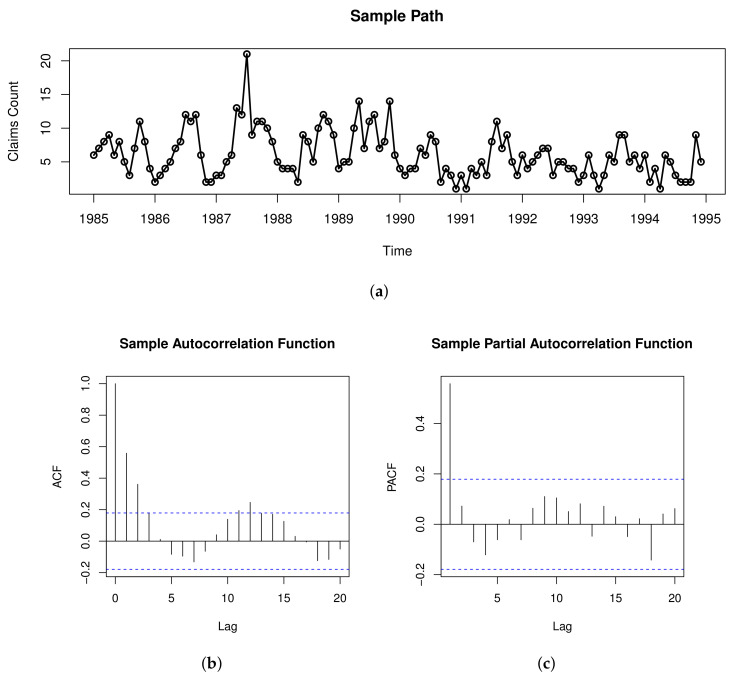
The sample path plot (**a**), ACF and PACF plots (**b**,**c**) for the counts of claimants.

In this section, we use the PSETINAR${\left(2;1,1\right)}_{T}$ process to fit the series of monthly counts of claimants collecting short-term disability benefits. In the dataset, all the claimants are male, have cuts, lacerations or punctures, and are between the ages of 35 and 54. In addition, they all work in the logging industry and collect benefits from the Workers’ Compensation Board (WCB) of British Columbia. The dataset consists of 120 observations, from 1985 to 1994 (Freeland [36]). The simulations were performed on the R3.6.2 software. The threshold vector was calculated by the algorithms (the three-step algorithm of NeSS combined with quasi-likelihood principle and the algorithm of NeSS combined with least squares principle) described in Section 3.2. We uses the command *constrOptim* to optimize the objective function of the maximum likelihood estimation. Figure 4 shows the sample path, ACF and PACF plots of the observations. It can be seen from Figure 4 that this dataset is a dependent counting time series with periodic characteristic.

**Figure 5:**
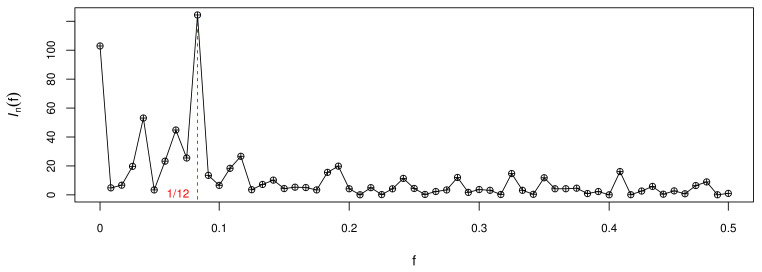
The periodogram plot for the monthly counts of claimants.

We use the periodogram method to determine the period about this dataset and draw Figure 5, from which it can be seen that $I_{n}\left(f_{k}\right)$ reach maximum at $f_{k}=1/12$, and concluded that T=12. This displays the periodic characteristic of the data and exhibits a form of periodic change per year.

Table 21 displays the descriptive statistics for the monthly counts of claimants collecting short-term disability benefits from WCB. From Table 21, we can see that the mean and variance are approximately equal in some months. We can assume that the distribution of the innovations is a periodic Poisson. However, some months and the total data indicate overdispersion. We find that the dataset has no zero and the minimum value is one. This leads us to consider the periodic Poisson, periodic Geometric, zero-truncated periodic Poisson and zero-truncated periodic Geometric distributions for the innovations to fit the model, respectively. Before the model fitting, we first estimate the threshold vector. The ${\hat{r}}_{CLS}$ is calculated by (9) and the ${\hat{r}}_{MQL}$ is calculated through (10) by using the three-step algorithm. Table 22 summarizes the fitting results of ${\hat{r}}_{CLS}$ and ${\hat{r}}_{MQL}$. Due to the lesser data, to fit the model better, when the number of data in each regime is relatively smaller than two or the threshold is the maximum or minimum value of the boundary, we think that these monthly data do not have a piecewise phenomenon, that is, March, July, and August do not have piecewise phenomena.

To capture the piecewise phenomenon of this time series dataset, we use PINAR${\left(1\right)}_{T}$ and PSETINAR${\left(2;1,1\right)}_{T}$ models with period T=12 to fit the dataset, respectively. The PINAR(1) process proposed by Monteiro et al. [22] with the following recursive equation

(12) $$X_{t}=\alpha_{t}\circ X_{t-1}+Z_{t},$$

with $\alpha_{t}=\alpha_{j}\in \left(0,1\right)$ for $t=j+sT(j=1,\cdots ,T,s\in N_{0})$, the definition of thinning operator “∘" and innovation process $\left\{Z_{t}\right\}$ is the same as the PSETINAR${\left(2;1,1\right)}_{T}$ process.

It is worth mentioning that for this dataset, the conditional least squares and quasi-likelihood methods produce non-admissible estimators for some months, so we use the conditional maximum likelihood approach to estimate the parameters. Next, we use PSETINAR${\left(2;1,1\right)}_{12}$ and PINAR${\left(1\right)}_{12}$ models to fit the dataset in combination with the four innovation distributions mentioned before. Here, the threshold vectors are based on ${\hat{r}}_{MQL}$. The AIC and BIC are listed in Table 23. When we fit the dataset, we hope to get smaller AIC and BIC values. From the results in Table 23, we can conclude that the PSETINAR${\left(2;1,1\right)}_{12}$ model with zero-truncated periodic Poisson distribution is more suitable. Then, we do the conditional maximum likelihood estimation, and the results are listed in Table 24. Some estimators of the parameters in Table 24, for example, the $\alpha^{\left(2\right)}$ of January, May, June, September, October and November, are not statistically significant, suggesting that on those months, the number of claims is mainly modeled through the innovation process.

To check the predictability of the PSETINAR${\left(2;1,1\right)}_{T}$ model, we carry out the *h*-step-ahead forecasting for varying *h* of the PSETINAR${\left(2;1,1\right)}_{T}$ model. The *h*-step-ahead conditional expectation point predictor of the PSETINAR${\left(2;1,1\right)}_{T}$ model is given by

$${\hat{X}}_{j+sT+h}=E\left[X_{j+sT+h}|X_{j+sT}\right],\;h=1,2,\cdots .$$

Specifically, the one-step-ahead conditional expectation point predictor is given by

$${\hat{X}}_{j+sT+1}=E\left[X_{j+sT+1}|X_{j+sT}\right]=\alpha_{j+1}^{\left(1\right)}X_{j+sT}I_{j+sT}^{\left(1\right)}+\alpha_{j+1}^{\left(2\right)}X_{j+sT}I_{j+sT}^{\left(2\right)}+\lambda_{j+1}.$$

However, the conditional expectation will seldom produce integer-valued forecasts. Recently, coherent forecasting techniques have been recommended, which only produce forecasts in $N_{0}$. This is achieved by computing the *h*-step-ahead forecasting conditional distribution. As pointed out by Möller et al. [37], this approach leads to forecasts themselves being easily obtained from the median or the mode of the forecasting distribution. In addition, Li et al. [38] and Kang et al. [8] have applied this method to forecast the integer-valued processes. Homburg et al. [39] discussed the prediction methods based on conditional distributions and Gaussian approximations and applied them to some integer-valued processes and compared them. For the PSETINAR${\left(2;1,1\right)}_{T}$ process, the one-step-ahead conditional distribution of $X_{j+sT+1}$ given $X_{j+sT}$ is given by

PXj+sT+1=xj+sT+1|Xj+sT=xj+sT=∑i=1minxj+sT,xj+sT+1∑k=12xj+sTiαj+1ki1−αj+1kxj+sT−iIj+sTkPZj+sT+1=xj+sT+1−i.

Due to the existence of the threshold, while we use the conditional expectation method to predict $X_{j+sT+h},h>1$, we have to predict the previous moment of $X_{j+sT+h-1}$ first and compare it with the corresponding threshold before we do the next prediction. We do the same for the conditional distribution method. (To prevent confusion, we call this method a point-wise conditional distribution forecast. The predictors completely based on *h*-step-ahead conditional distribution without intermediate step prediction will be discussed later.) The mode of *h*-step-ahead point-wise conditional distribution can be viewed as the point prediction. Here we compare the two forecasting methods, a standard descriptive measure of forecasting accuracy, namely, *h*-step-ahead predicted root mean squared error (PRMSE) is adopted. This measure can be given by

$$PRMSE=\sqrt{\frac{1}{K-K_{0}}\sum_{t=K_{0}+1}^{K}{\left(X_{t+h}-{\hat{X}}_{t+h}\right)}^{2}},\;h=1,2,\cdots ,$$

where *K* is the full sample size, we split the data into two parts, and the last $K-K_{0}$ observations as a forecasting evaluation sample. We forecast the value of the last year when h=1,2,3,12.

The PRMSEs of the *h*-step-ahead point predictors are list in Table 25. For conditional expectation point predictors, the PRMSEs of PSETINAR${\left(2;1,1\right)}_{12}$ with zero-truncated periodic Poisson distribution are smaller than the PINAR${\left(1\right)}_{12}$ with periodic Poisson and zero-truncated periodic Poisson distributions. This further shows the superiority of our model. The PRMSEs of the one-step-ahead point predictors are smaller than others. This is very natural because we use the value of the previous moment as the explanatory variable. For PSETINAR${\left(2;1,1\right)}_{12}$ with zero-truncated periodic Poisson distribution, the PRMSEs of twelve-step-ahead predictors are smaller than other *h*-step-ahead predictors except for one-step-ahead. This may be because our period is 12. The PRMSE of one-step-ahead conditional expectation point predictors is smaller than point-wise conditional distribution point predictors. Thus, the former method is better for this dataset.

**Figure 6:**
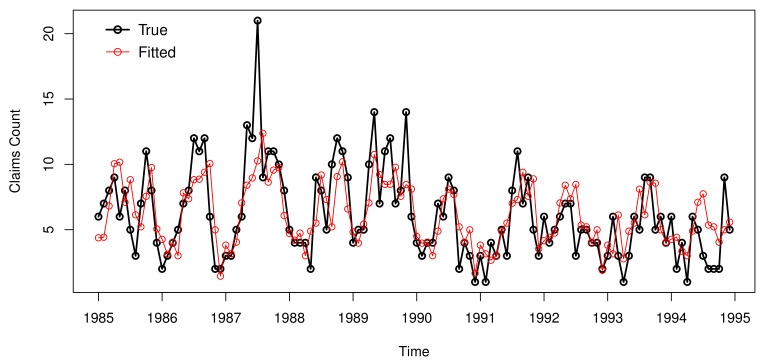
Plot of fitted curves of the claims data.

The PRMSEs of the one-step-ahead fitted series calculated by conditional expectation and conditional distribution are 2.434 and 3.565, respectively. This further illustrates that for our dataset, one-step-ahead forecasting conditional expectation is better than conditional distribution. The original data and the fitted series (calculated by the one-step-ahead conditional expectation based on the observations of the previous moments) by the PSETINAR${\left(2;1,1\right)}_{12}$ model with zero-truncated periodic Poisson distribution are plotted in Figure 6. It is observed that the trend is similar to the original data. Except for the points with large value (the unexpected prediction may be due to the wrong judgement of regime), this model fits the data well.

**Figure 7:**
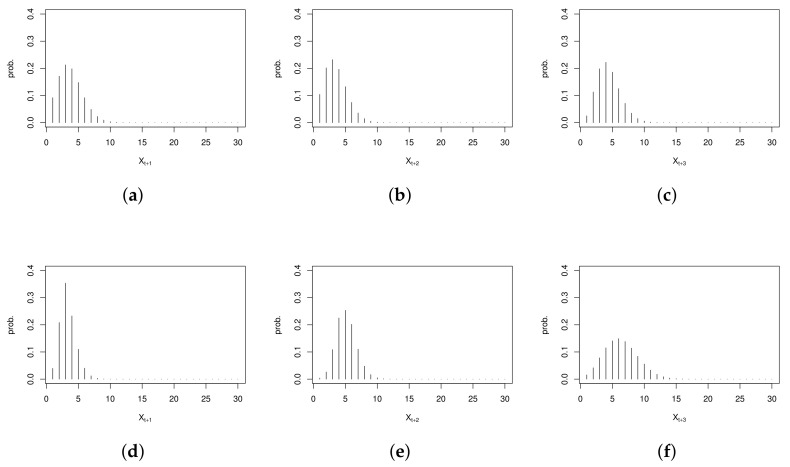
The *h*-step-ahead forecasting conditional distribution for the counts of claimants: (**a**–**c**) conditional on the count of claimants in December 1993; (**d**–**f**) conditional on the count of claimants in February 1994.

Actually, we can get the *h*-step-ahead conditional distribution; here, we list the two-step-ahead and three-step-ahead conditional distributions as an example,

$$\begin{array}{cc} \; & P\left(X_{j+sT+2}=x_{j+sT+2}|X_{j+sT}=x_{j+sT}\right)\\ = & \sum_{m=0}^{n}P\left(X_{j+sT+1}=m|X_{j+sT}=x_{j+sT}\right)P\left(X_{j+sT+2}=x_{j+sT+2}|X_{j+sT+1}=m\right), \end{array}$$

and

$$\begin{array}{cc} \; & P\left(X_{j+sT+3}=x_{j+sT+3}|X_{j+sT}=x_{j+sT}\right)\\ = & \sum_{m=0}^{n}P\left(X_{j+sT+2}=m|X_{j+sT}=x_{j+sT}\right)P\left(X_{j+sT+3}=x_{j+sT+3}|X_{j+sT+2}=m\right), \end{array}$$

where m∈{0,1,⋯,n} is the possible domain of $X_{j+sT}$, j=1,⋯,T, and $s\in N_{0}$. When h=1,2,3, we show the plots of the *h*-step-ahead conditional distribution in Figure 7, where $x_{j+sT}$ represents the count of claimants in December 1993 and February 1994, respectively. The mode of h-step-ahead conditional distribution can be viewed as the point prediction. The PRMSEs of the two-step-ahead and three-step-ahead point predictors for the last year are 3.227 and 3.215, respectively, which is larger than the point-wise conditional distribution method described before. Maybe for other datasets or models, the *h*-step-ahead forecasting conditional distribution will show some advantages. We will not go into details here.

## 6. Conclusions

This paper extended the PSETINAR${\left(2;1,1\right)}_{T}$ process proposed by Pereira et al. [25], by removing the assumption of Poisson distribution of $\left\{Z_{t}\right\}$ and considered the PSETINAR ${\left(2;1,1\right)}_{T}$ process under weak conditions that the second moment of $\left\{Z_{t}\right\}$ is finite. The ergodicity of the process is established. MQL-estimators of the model parameters vector β, MQL-estimators and CLS-estimators of the thresholds vector r are obtained. Moreover, through simulation, we can see the advantages of the quasi-likelihood method by comparing with the conditional maximum likelihood and conditional least square methods. An application to a real dataset is presented. In addition, the forecasting problem of this dataset is addressed.

In this paper, we only discuss the PSETINAR${\left(2;1,1\right)}_{T}$ process for univariate time series. Hence, an extension for the multivariate PSETINAR${\left(2;1,1\right)}_{T}$ process with a diagonal or cross-correlation autoregressive matrix is a topic for future investigation. Furthermore, it is also important to stress that beyond this extension, there are a number of interesting problems for future research in this area. For example, even a simple periodic model can have an inordinately large number of parameters. This is also true for PSETINAR${\left(2;1,1\right)}_{T}$ models and even multi-period models. Therefore, the development of procedures of dimensionality reduction to overcome the computational difficulties is an impending problem. This remains a topic of future research.

## Figures and Tables

**Table 1:** Bias and MSE for Series A of Model I (MSE in parentheses): CLS, MQL and CML.

*N*	Method	$\alpha_{1}^{\left(1\right)}$	$\alpha_{1}^{\left(2\right)}$	$\lambda_{1}$	$\alpha_{2}^{\left(1\right)}$	$\alpha_{2}^{\left(2\right)}$	$\lambda_{2}$	$\alpha_{3}^{\left(1\right)}$	$\alpha_{3}^{\left(2\right)}$	$\lambda_{3}$
50	CLS	0.001	−0.001	0.001	−0.018	−0.004	0.006	0.008	0.005	−0.025
		(0.052)	(0.014)	(0.253)	(0.131)	(0.024)	(0.230)	(0.160)	(0.024)	(0.326)
	MQL	0.000	−0.002	0.006	−0.015	−0.004	0.002	0.011	0.006	−0.030
		(0.054)	(0.014)	(0.266)	(0.126)	(0.023)	(0.220)	(0.156)	(0.024)	(0.316)
	CML	0.024	0.010	−0.047	0.054	0.019	−0.079	0.003	0.007	−0.027
		(0.024)	(0.008)	(0.117)	(0.062)	(0.016)	(0.126)	(0.047)	(0.013)	(0.134)
100	CLS	0.004	0.000	−0.006	0.013	−0.001	−0.005	0.002	−0.003	0.008
		(0.026)	(0.007)	(0.132)	(0.058)	(0.011)	(0.108)	(0.085)	(0.012)	(0.168)
	MQL	0.004	0.000	−0.006	0.013	−0.001	−0.006	−0.001	−0.004	0.012
		(0.024)	(0.007)	(0.120)	(0.057)	(0.011)	(0.105)	(0.082)	(0.011)	(0.162)
	CML	0.012	0.004	−0.023	0.036	0.007	−0.034	0.003	0.000	−0.001
		(0.014)	(0.004)	(0.067)	(0.036)	(0.008)	(0.073)	(0.024)	(0.006)	(0.066)
300	CLS	−0.003	−0.002	0.009	0.002	0.000	−0.005	−0.002	0.000	−0.001
		(0.010)	(0.003)	(0.051)	(0.020)	(0.004)	(0.034)	(0.028)	(0.004)	(0.055)
	MQL	−0.002	−0.001	0.007	0.001	0.000	−0.004	−0.003	0.000	0.000
		(0.009)	(0.002)	(0.045)	(0.019)	(0.004)	(0.033)	(0.027)	(0.003)	(0.053)
	CML	0.000	0.000	0.000	0.003	0.001	−0.007	0.001	0.002	−0.006
		(0.005)	(0.001)	(0.025)	(0.014)	(0.003)	(0.024)	(0.007)	(0.002)	(0.020)

**Table 2:** Bias and MSE for Series B of Model I (MSE in parentheses): CLS, MQL and CML.

*N*	Method	$\alpha_{1}^{\left(1\right)}$	$\alpha_{1}^{\left(2\right)}$	$\lambda_{1}$	$\alpha_{2}^{\left(1\right)}$	$\alpha_{2}^{\left(2\right)}$	$\lambda_{2}$	$\alpha_{3}^{\left(1\right)}$	$\alpha_{3}^{\left(2\right)}$	$\lambda_{3}$
50	CLS	0.009	0.001	0.003	0.014	0.003	−0.015	−0.013	−0.009	0.032
		(0.119)	(0.015)	(0.238)	(0.166)	(0.031)	(0.365)	(0.105)	(0.026)	(0.525)
	MQL	0.010	0.001	0.003	0.013	0.003	−0.014	−0.012	−0.009	0.031
		(0.129)	(0.015)	(0.241)	(0.161)	(0.030)	(0.354)	(0.104)	(0.026)	(0.516)
	CML	0.006	0.003	0.001	0.014	0.006	−0.020	0.008	0.003	−0.019
		(0.043)	(0.007)	(0.090)	(0.062)	(0.016)	(0.150)	(0.045)	(0.014)	(0.229)
100	CLS	0.007	0.000	−0.001	−0.022	−0.009	0.042	−0.004	−0.002	0.003
		(0.061)	(0.008)	(0.133)	(0.076)	(0.014)	(0.173)	(0.046)	(0.012)	(0.222)
	MQL	0.008	0.000	−0.003	−0.023	−0.010	0.044	−0.004	−0.002	0.003
		(0.055)	(0.007)	(0.116)	(0.076)	(0.014)	(0.172)	(0.045)	(0.012)	(0.216)
	CML	0.002	0.000	−0.001	−0.004	−0.001	0.013	0.000	0.001	−0.008
		(0.018)	(0.003)	(0.040)	(0.031)	(0.008)	(0.078)	(0.027)	(0.007)	(0.127)
300	CLS	0.003	0.000	−0.003	0.002	0.000	0.002	−0.003	−0.001	−0.001
		(0.020)	(0.003)	(0.043)	(0.026)	(0.005)	(0.060)	(0.017)	(0.004)	(0.081)
	MQL	0.003	−0.001	−0.002	0.001	0.000	0.004	−0.002	0.000	−0.004
		(0.019)	(0.002)	(0.039)	(0.025)	(0.005)	(0.058)	(0.016)	(0.004)	(0.077)
	CML	−0.002	−0.002	0.003	0.003	0.001	−0.001	−0.003	0.000	−0.002
		(0.006)	(0.001)	(0.014)	(0.009)	(0.002)	(0.025)	(0.009)	(0.003)	(0.043)

**Table 3:** Bias and MSE for Series C of Model I (MSE in parentheses): CLS, MQL and CML.

*N*	Method	$\alpha_{1}^{\left(1\right)}$	$\alpha_{1}^{\left(2\right)}$	$\lambda_{1}$	$\alpha_{2}^{\left(1\right)}$	$\alpha_{2}^{\left(2\right)}$	$\lambda_{2}$	$\alpha_{3}^{\left(1\right)}$	$\alpha_{3}^{\left(2\right)}$	$\lambda_{3}$
50	CLS	−0.013	−0.010	0.146	−0.010	−0.003	0.053	−0.010	−0.007	0.054
		(0.022)	(0.011)	(2.088)	(0.082)	(0.022)	(1.915)	(0.078)	(0.026)	(3.823)
	MQL	−0.010	−0.008	0.117	−0.010	−0.003	0.052	−0.014	−0.009	0.079
		(0.022)	(0.010)	(2.000)	(0.082)	(0.021)	(1.913)	(0.075)	(0.025)	(3.709)
	CML	0.003	0.001	−0.015	0.044	0.021	−0.201	0.003	0.000	−0.033
		(0.012)	(0.006)	(1.119)	(0.044)	(0.013)	(1.054)	(0.025)	(0.010)	(1.286)
100	CLS	0.001	−0.002	0.015	−0.003	0.001	0.013	0.002	−0.003	0.022
		(0.014)	(0.006)	(1.323)	(0.043)	(0.011)	(1.046)	(0.038)	(0.012)	(1.772)
	MQL	0.000	−0.003	0.034	−0.002	0.001	0.008	0.001	−0.003	0.027
		(0.012)	(0.006)	(1.203)	(0.042)	(0.011)	(1.027)	(0.037)	(0.012)	(1.726)
	CML	0.006	0.001	−0.029	0.018	0.010	−0.085	0.011	0.003	−0.043
		(0.007)	(0.003)	(0.672)	(0.026)	(0.007)	(0.657)	(0.012)	(0.005)	(0.620)
300	CLS	0.000	0.000	0.006	0.002	0.002	−0.014	0.006	0.003	−0.040
		(0.006)	(0.003)	(0.586)	(0.014)	(0.004)	(0.350)	(0.013)	(0.004)	(0.606)
	MQL	0.001	0.000	0.002	0.001	0.001	−0.010	0.005	0.002	−0.032
		(0.005)	(0.002)	(0.527)	(0.014)	(0.004)	(0.341)	(0.012)	(0.004)	(0.589)
	CML	0.002	0.001	−0.013	0.005	0.003	−0.030	0.003	0.002	−0.026
		(0.003)	(0.001)	(0.262)	(0.011)	(0.003)	(0.267)	(0.004)	(0.002)	(0.201)

**Table 4:** Bias and MSE for Series A of Model II (MSE in parentheses): CLS, MQL and CML.

*N*	Method	$\alpha_{1}^{\left(1\right)}$	$\alpha_{1}^{\left(2\right)}$	$\lambda_{1}$	$\alpha_{2}^{\left(1\right)}$	$\alpha_{2}^{\left(2\right)}$	$\lambda_{2}$	$\alpha_{3}^{\left(1\right)}$	$\alpha_{3}^{\left(2\right)}$	$\lambda_{3}$
50	CLS	−0.013	−0.005	0.021	−0.016	−0.014	0.024	−0.025	−0.011	0.044
		(0.073)	(0.011)	(0.247)	(0.291)	(0.032)	(0.408)	(0.330)	(0.024)	(0.449)
	MQL	−0.011	−0.005	0.019	−0.012	−0.013	0.019	−0.020	−0.010	0.040
		(0.067)	(0.011)	(0.228)	(0.287)	(0.032)	(0.402)	(0.330)	(0.024)	(0.439)
	CML	0.014	0.005	−0.026	0.041	0.011	−0.050	0.004	0.008	−0.016
		(0.016)	(0.005)	(0.076)	(0.040)	(0.010)	(0.158)	(0.020)	(0.007)	(0.153)
100	CLS	0.003	0.003	−0.011	−0.013	−0.011	0.027	−0.002	0.001	−0.005
		(0.032)	(0.005)	(0.116)	(0.145)	(0.016)	(0.195)	(0.170)	(0.012)	(0.219)
	MQL	0.001	0.002	−0.006	−0.011	−0.010	0.024	−0.001	0.001	−0.006
		(0.030)	(0.005)	(0.104)	(0.143)	(0.016)	(0.194)	(0.169)	(0.011)	(0.215)
	CML	0.006	0.004	−0.014	0.020	0.006	−0.021	0.005	0.004	−0.019
		(0.007)	(0.002)	(0.039)	(0.022)	(0.005)	(0.080)	(0.011)	(0.003)	(0.072)
300	CLS	0.001	0.000	−0.005	−0.003	0.000	0.009	−0.001	0.000	−0.001
		(0.011)	(0.002)	(0.039)	(0.050)	(0.006)	(0.067)	(0.052)	(0.004)	(0.077)
	MQL	0.000	0.000	−0.005	−0.003	−0.001	0.010	0.000	0.000	−0.002
		(0.010)	(0.001)	(0.034)	(0.049)	(0.006)	(0.067)	(0.052)	(0.004)	(0.076)
	CML	0.005	0.001	−0.011	0.000	0.004	0.000	0.002	0.002	−0.007
		(0.003)	(0.001)	(0.013)	(0.008)	(0.002)	(0.026)	(0.004)	(0.001)	(0.026)

**Table 5:** Bias and MSE for Series B of Model II (MSE in parentheses): CLS, MQL and CML.

*N*	Method	$\alpha_{1}^{\left(1\right)}$	$\alpha_{1}^{\left(2\right)}$	$\lambda_{1}$	$\alpha_{2}^{\left(1\right)}$	$\alpha_{2}^{\left(2\right)}$	$\lambda_{2}$	$\alpha_{3}^{\left(1\right)}$	$\alpha_{3}^{\left(2\right)}$	$\lambda_{3}$
50	CLS	0.009	0.003	−0.019	0.005	−0.012	0.016	0.006	−0.003	−0.002
		(0.038)	(0.007)	(2.068)	(0.382)	(0.043)	(5.495)	(0.217)	(0.026)	(5.702)
	MQL	0.008	0.003	−0.017	−0.055	−0.022	0.070	0.009	−0.002	−0.008
		(0.037)	(0.006)	(1.995)	(0.378)	(0.043)	(5.461)	(0.220)	(0.026)	(5.718)
	CML	0.007	0.004	−0.017	0.015	0.004	−0.025	0.014	0.007	−0.031
		(0.005)	(0.002)	(0.590)	(0.025)	(0.006)	(1.380)	(0.008)	(0.004)	(1.326)
100	CLS	−0.001	−0.002	0.007	−0.006	−0.004	0.007	−0.006	−0.002	0.011
		(0.019)	(0.003)	(1.143)	(0.190)	(0.023)	(3.017)	(0.114)	(0.011)	(2.871)
	MQL	0.000	−0.002	0.004	−0.005	−0.004	0.007	−0.006	−0.003	0.012
		(0.018)	(0.003)	(1.091)	(0.189)	(0.023)	(3.001)	(0.115)	(0.012)	(2.882)
	CML	0.006	0.002	−0.012	0.008	0.004	−0.017	0.001	0.007	−0.017
		(0.002)	(0.001)	(0.238)	(0.012)	(0.003)	(0.691)	(0.004)	(0.002)	(0.660)
300	CLS	−0.003	−0.001	0.004	−0.004	−0.001	−0.006	0.003	−0.002	−0.006
		(0.006)	(0.001)	(0.361)	(0.062)	(0.007)	(0.889)	(0.033)	(0.004)	(0.848)
	MQL	−0.003	0.000	0.003	−0.002	0.000	−0.008	0.004	−0.002	−0.007
		(0.006)	(0.001)	(0.345)	(0.062)	(0.007)	(0.887)	(0.033)	(0.004)	(0.849)
	CML	0.000	0.001	−0.001	0.001	0.001	−0.011	0.004	0.002	−0.015
		(0.001)	(0.000)	(0.069)	(0.004)	(0.001)	(0.205)	(0.001)	(0.001)	(0.222)

**Table 6:** Bias and MSE for Series C of Model II (MSE in parentheses): CLS, MQL and CML.

*N*	Method	$\alpha_{1}^{\left(1\right)}$	$\alpha_{1}^{\left(2\right)}$	$\lambda_{1}$	$\alpha_{2}^{\left(1\right)}$	$\alpha_{2}^{\left(2\right)}$	$\lambda_{2}$	$\alpha_{3}^{\left(1\right)}$	$\alpha_{3}^{\left(2\right)}$	$\lambda_{3}$
50	CLS	−0.004	−0.002	0.069	−0.019	−0.008	0.061	−0.011	−0.008	0.131
		(0.038)	(0.007)	(2.068)	(0.382)	(0.043)	(5.495)	(0.217)	(0.026)	(5.702)
	MQL	−0.004	−0.002	0.067	−0.016	−0.007	0.051	−0.009	−0.007	0.122
		(0.037)	(0.006)	(1.995)	(0.378)	(0.043)	(5.461)	(0.220)	(0.026)	(5.718)
	CML	0.010	0.005	−0.019	0.037	0.014	−0.152	0.013	0.009	−0.038
		(0.005)	(0.002)	(0.590)	(0.025)	(0.006)	(1.380)	(0.008)	(0.004)	(1.326)
100	CLS	0.000	0.000	−0.005	−0.020	−0.004	0.054	0.001	−0.008	0.046
		(0.019)	(0.003)	(1.143)	(0.190)	(0.023)	(3.017)	(0.114)	(0.011)	(2.871)
	MQL	−0.002	−0.001	0.006	−0.020	−0.004	0.054	0.002	−0.008	0.045
		(0.018)	(0.003)	(1.091)	(0.189)	(0.023)	(3.001)	(0.115)	(0.012)	(2.882)
	CML	0.008	0.003	−0.059	0.016	0.005	−0.068	0.009	0.003	−0.047
		(0.002)	(0.001)	(0.238)	(0.012)	(0.003)	(0.691)	(0.004)	(0.002)	(0.660)
300	CLS	0.000	−0.001	−0.007	−0.005	−0.001	0.010	−0.014	−0.004	0.071
		(0.006)	(0.001)	(0.361)	(0.062)	(0.007)	(0.889)	(0.033)	(0.004)	(0.848)
	MQL	0.000	−0.001	−0.008	−0.005	−0.001	0.011	−0.014	−0.004	0.072
		(0.006)	(0.001)	(0.345)	(0.062)	(0.007)	(0.887)	(0.033)	(0.004)	(0.849)
	CML	0.000	0.000	−0.012	0.005	0.001	−0.020	0.004	0.002	−0.021
		(0.001)	(0.000)	(0.069)	(0.004)	(0.001)	(0.205)	(0.001)	(0.001)	(0.222)

**Table 7:** Bias and MSE for Series A of Model III with *N* = 300 (MSE in parentheses).

ρ	Method	$\alpha_{1}^{\left(1\right)}$	$\alpha_{1}^{\left(2\right)}$	$\lambda_{1}$	$\alpha_{2}^{\left(1\right)}$	$\alpha_{2}^{\left(2\right)}$	$\lambda_{2}$	$\alpha_{3}^{\left(1\right)}$	$\alpha_{3}^{\left(2\right)}$	$\lambda_{3}$
(0.9, 0.9, 0.9)	CLS	0.002	0.002	−0.004	0.009	0.004	−0.014	−0.007	0.000	0.002
		(0.010)	(0.002)	(0.049)	(0.022)	(0.004)	(0.041)	(0.026)	(0.004)	(0.055)
	MQL	0.002	0.002	−0.004	0.009	0.004	−0.014	−0.007	−0.001	0.003
		(0.009)	(0.002)	(0.042)	(0.021)	(0.004)	(0.040)	(0.026)	(0.004)	(0.053)
	CML	−0.021	−0.009	0.046	−0.043	−0.018	0.057	−0.055	−0.022	0.081
		(0.006)	(0.001)	(0.027)	(0.013)	(0.003)	(0.030)	(0.012)	(0.003)	(0.034)
(0.8, 0.8, 0.8)	CLS	−0.001	−0.001	0.000	0.005	−0.004	0.005	−0.005	−0.004	0.012
		(0.010)	(0.002)	(0.048)	(0.026)	(0.004)	(0.044)	(0.030)	(0.004)	(0.056)
	MQL	−0.001	−0.001	0.000	0.005	−0.004	0.006	−0.008	−0.005	0.016
		(0.009)	(0.002)	(0.042)	(0.026)	(0.004)	(0.043)	(0.030)	(0.004)	(0.054)
	CML	−0.042	−0.018	0.088	−0.080	−0.040	0.122	−0.121	−0.049	0.183
		(0.007)	(0.002)	(0.033)	(0.015)	(0.004)	(0.041)	(0.028)	(0.004)	(0.067)

**Table 8:** Bias and MSE for Series B of Model III with *N* = 300 (MSE in parentheses).

ρ	Method	$\alpha_{1}^{\left(1\right)}$	$\alpha_{1}^{\left(2\right)}$	$\lambda_{1}$	$\alpha_{2}^{\left(1\right)}$	$\alpha_{2}^{\left(2\right)}$	$\lambda_{2}$	$\alpha_{3}^{\left(1\right)}$	$\alpha_{3}^{\left(2\right)}$	$\lambda_{3}$
(0.9, 0.9, 0.9)	CLS	0.003	0.001	−0.001	0.001	0.000	0.000	0.004	0.000	−0.006
		(0.020)	(0.003)	(0.041)	(0.031)	(0.005)	(0.068)	(0.018)	(0.004)	(0.083)
	MQL	0.002	0.001	0.001	0.001	−0.001	0.001	0.003	−0.001	−0.003
		(0.018)	(0.002)	(0.036)	(0.030)	(0.005)	(0.065)	(0.017)	(0.004)	(0.080)
	CML	−0.023	−0.009	0.041	−0.080	−0.033	0.122	−0.065	−0.030	0.140
		(0.007)	(0.001)	(0.018)	(0.019)	(0.004)	(0.050)	(0.014)	(0.003)	(0.069)
(0.8, 0.8, 0.8)	CLS	0.001	0.001	−0.006	−0.005	−0.005	0.017	−0.002	−0.002	0.009
		(0.023)	(0.003)	(0.045)	(0.033)	(0.006)	(0.070)	(0.018)	(0.004)	(0.083)
	MQL	0.002	0.002	−0.008	−0.004	−0.005	0.016	−0.002	−0.002	0.010
		(0.021)	(0.002)	(0.039)	(0.032)	(0.005)	(0.067)	(0.017)	(0.004)	(0.078)
	CML	−0.043	−0.015	0.064	−0.156	−0.065	0.240	−0.122	−0.054	0.263
		(0.009)	(0.001)	(0.021)	(0.040)	(0.008)	(0.104)	(0.023)	(0.005)	(0.119)

**Table 9:** Bias and MSE for Series C of Model III with *N* = 300 (MSE in parentheses).

ρ	Method	$\alpha_{1}^{\left(1\right)}$	$\alpha_{1}^{\left(2\right)}$	$\lambda_{1}$	$\alpha_{2}^{\left(1\right)}$	$\alpha_{2}^{\left(2\right)}$	$\lambda_{2}$	$\alpha_{3}^{\left(1\right)}$	$\alpha_{3}^{\left(2\right)}$	$\lambda_{3}$
(0.9, 0.9, 0.9)	CLS	0.003	0.001	−0.024	−0.014	−0.007	0.078	−0.002	−0.002	0.021
		(0.006)	(0.002)	(0.534)	(0.020)	(0.005)	(0.485)	(0.014)	(0.004)	(0.643)
	MQL	0.003	0.001	−0.020	−0.013	−0.007	0.077	−0.001	−0.002	0.018
		(0.005)	(0.002)	(0.472)	(0.020)	(0.005)	(0.484)	(0.014)	(0.004)	(0.630)
	CML	−0.044	−0.028	0.432	−0.122	−0.064	0.631	−0.186	−0.097	1.250
		(0.005)	(0.002)	(0.470)	(0.020)	(0.006)	(0.595)	(0.044)	(0.013)	(2.143)
(0.8, 0.8, 0.8)	CLS	0.001	−0.001	−0.003	−0.008	−0.003	0.036	0.000	−0.001	0.005
		(0.005)	(0.002)	(0.448)	(0.023)	(0.005)	(0.494)	(0.016)	(0.004)	(0.668)
	MQL	0.000	−0.001	0.002	−0.008	−0.002	0.034	−0.001	−0.002	0.007
		(0.005)	(0.002)	(0.407)	(0.023)	(0.005)	(0.490)	(0.015)	(0.004)	(0.661)
	CML	−0.074	−0.045	0.706	−0.158	−0.085	0.811	−0.296	−0.144	1.907
		(0.008)	(0.003)	(0.754)	(0.027)	(0.009)	(0.800)	(0.098)	(0.024)	(4.230)

**Table 10:** Bias, bias median and MSE for Series A of Model I.

*N*	Para.		MQL				CLS	
		Bias	Median	MSE		Bias	Median	MSE
50	$r_{1}$	−0.167	0	0.447		0.042	0	0.550
	$r_{2}$	0.422	0	1.986		0.723	0	2.841
	$r_{3}$	0.457	0	1.975		0.947	0	3.779
100	$r_{1}$	−0.107	0	0.151		−0.003	0	0.137
	$r_{2}$	0.224	0	1.378		0.570	0	2.428
	$r_{3}$	0.245	0	0.861		0.505	0	1.903
300	$r_{1}$	−0.007	0	0.007		0.000	0	0.002
	$r_{2}$	0.027	0	0.283		0.117	0	0.477
	$r_{3}$	0.021	0	0.035		0.066	0	0.200

**Table 11:** Bias, bias median and MSE for Series B of Model I.

*N*	Para.		MQL				CLS	
		Bias	Median	MSE		Bias	Median	MSE
50	$r_{1}$	0.499	0	2.129		1.294	1	4.176
	$r_{2}$	0.538	0	2.320		0.868	0	3.142
	$r_{3}$	0.139	0	2.687		0.634	0	3.610
100	$r_{1}$	0.555	1	1.933		1.301	1	3.597
	$r_{2}$	0.283	0	1.437		0.643	0	2.473
	$r_{3}$	0.107	0	2.537		0.599	0	3.431
300	$r_{1}$	0.480	1	1.518		1.215	1	2.485
	$r_{2}$	0.021	0	0.213		0.141	0	0.489
	$r_{3}$	−0.095	0	1.191		0.261	0	1.825

**Table 12:** Bias, bias median and MSE for Series C of Model I.

*N*	Para.		MQL				CLS	
		Bias	Median	MSE		Bias	Median	MSE
50	$r_{1}$	−0.012	0	0.588		0.023	0	0.661
	$r_{2}$	0.268	0	5.378		0.541	0	5.909
	$r_{3}$	0.155	0	1.433		0.216	0	1.750
100	$r_{1}$	0.015	0	0.079		0.023	0	0.081
	$r_{2}$	0.072	0	2.332		0.254	0	2.972
	$r_{3}$	0.041	0	0.325		0.050	0	0.330
300	$r_{1}$	0.000	0	0.000		0.000	0	0.000
	$r_{2}$	−0.015	0	0.317		0.027	0	0.457
	$r_{3}$	0.002	0	0.004		0.002	0	0.004

**Table 13:** Bias, bias median and MSE for Series A of Model II.

*N*	Para.		MQL				CLS	
		Bias	Median	MSE		Bias	Median	MSE
50	$r_{1}$	0.027	0	1.231		0.407	0	2.227
	$r_{2}$	1.025	0	4.897		1.293	1	6.051
	$r_{3}$	1.582	1	7.600		2.003	1	9.905
100	$r_{1}$	−0.013	0	0.489		0.185	0	0.723
	$r_{2}$	0.944	0	4.808		1.271	0	6.215
	$r_{3}$	1.539	0	8.391		2.005	1	11.269
300	$r_{1}$	−0.042	0	0.066		0.022	0	0.070
	$r_{2}$	0.652	0	3.560		0.940	0	5.088
	$r_{3}$	0.605	0	3.243		1.062	0	6.540

**Table 14:** Bias, bias median and MSE for Series B of Model II.

*N*	Para.		MQL				CLS	
		Bias	Median	MSE		Bias	Median	MSE
50	$r_{1}$	1.231	1	5.527		2.134	2	9.638
	$r_{2}$	1.307	1	6.063		1.633	1	7.439
	$r_{3}$	0.840	0	6.658		1.237	1	8.211
100	$r_{1}$	1.070	1	3.954		1.972	2	8.050
	$r_{2}$	1.208	0	5.772		1.561	1	7.375
	$r_{3}$	0.998	0	7.652		1.488	1	9.644
300	$r_{1}$	1.059	1	3.143		1.829	2	5.611
	$r_{2}$	0.717	0	3.465		1.031	0	4.961
	$r_{3}$	0.617	0	5.925		1.153	0	8.549

**Table 15:** Bias, bias median and MSE for Series C of Model II.

*N*	Para.		MQL				CLS	
		Bias	Median	MSE		Bias	Median	MSE
50	$r_{1}$	−1.066	0	11.494		−0.859	0	12.671
	$r_{2}$	0.006	0	18.430		0.149	0	19.137
	$r_{3}$	−0.337	−1	27.211		−0.206	−1	27.764
100	$r_{1}$	−0.130	0	4.220		0.078	0	5.250
	$r_{2}$	0.538	0	22.610		0.696	0	23.536
	$r_{3}$	0.241	0	26.911		0.386	0	28.340
300	$r_{1}$	−0.040	0	0.236		−0.016	0	0.262
	$r_{2}$	1.213	0	26.909		1.389	0	28.515
	$r_{3}$	0.794	0	19.586		0.961	0	21.521

**Table 16:** Bias and MSE for Series A of Model I with “burn in” samples (MSE in parentheses): CLS, MQL and CML.

*N*	Method	$\alpha_{1}^{\left(1\right)}$	$\alpha_{1}^{\left(2\right)}$	$\lambda_{1}$	$\alpha_{2}^{\left(1\right)}$	$\alpha_{2}^{\left(2\right)}$	$\lambda_{2}$	$\alpha_{3}^{\left(1\right)}$	$\alpha_{3}^{\left(2\right)}$	$\lambda_{3}$
50	CLS	−0.002	−0.008	0.012	−0.018	−0.012	0.029	0.001	0.004	−0.008
		(0.067)	(0.017)	(0.338)	(0.132)	(0.024)	(0.241)	(0.168)	(0.025)	(0.351)
	MQL	0.001	−0.006	0.006	−0.016	−0.012	0.027	0.002	0.004	−0.007
		(0.066)	(0.017)	(0.331)	(0.134)	(0.024)	(0.240)	(0.174)	(0.024)	(0.347)
	CML	0.032	0.008	−0.061	0.043	0.007	−0.044	0.000	0.004	−0.005
		(0.027)	(0.008)	(0.124)	(0.056)	(0.015)	(0.125)	(0.046)	(0.012)	(0.125)
100	CLS	−0.005	−0.006	0.014	−0.011	−0.005	0.012	−0.001	−0.002	0.011
		(0.030)	(0.009)	(0.153)	(0.063)	(0.011)	(0.106)	(0.081)	(0.012)	(0.166)
	MQL	−0.006	−0.006	0.017	−0.012	−0.006	0.013	0.000	−0.002	0.008
		(0.028)	(0.008)	(0.138)	(0.061)	(0.010)	(0.103)	(0.078)	(0.012)	(0.158)
	CML	0.006	−0.001	−0.010	0.025	0.006	−0.031	0.000	0.001	0.003
		(0.015)	(0.004)	(0.069)	(0.035)	(0.007)	(0.069)	(0.021)	(0.006)	(0.061)
300	CLS	−0.001	−0.002	0.002	−0.003	−0.001	0.009	0.002	0.001	0.000
		(0.010)	(0.003)	(0.052)	(0.019)	(0.004)	(0.034)	(0.024)	(0.004)	(0.050)
	MQL	0.000	−0.001	0.000	−0.003	−0.001	0.008	0.001	0.001	0.002
		(0.009)	(0.002)	(0.047)	(0.019)	(0.003)	(0.033)	(0.024)	(0.003)	(0.049)
	CML	0.001	−0.001	−0.003	0.003	0.001	0.001	0.001	0.001	0.000
		(0.006)	(0.002)	(0.027)	(0.015)	(0.003)	(0.025)	(0.007)	(0.002)	(0.021)

**Table 17:** Bias and MSE for Series A of Model II with “burn in” samples (MSE in parentheses): CLS, MQL and CML.

*N*	Method	$\alpha_{1}^{\left(1\right)}$	$\alpha_{1}^{\left(2\right)}$	$\lambda_{1}$	$\alpha_{2}^{\left(1\right)}$	$\alpha_{2}^{\left(2\right)}$	$\lambda_{2}$	$\alpha_{3}^{\left(1\right)}$	$\alpha_{3}^{\left(2\right)}$	$\lambda_{3}$
50	CLS	0.009	0.000	−0.017	0.023	−0.011	0.005	−0.011	−0.004	0.005
		(0.067)	(0.011)	(0.242)	(0.306)	(0.035)	(0.424)	(0.303)	(0.026)	(0.479)
	MQL	0.010	0.000	−0.017	0.028	−0.010	0.000	−0.018	−0.005	0.012
		(0.065)	(0.010)	(0.227)	(0.302)	(0.035)	(0.420)	(0.355)	(0.026)	(0.505)
	CML	0.022	0.006	−0.042	0.053	0.013	−0.048	0.007	0.007	−0.032
		(0.015)	(0.004)	(0.075)	(0.045)	(0.010)	(0.151)	(0.022)	(0.007)	(0.148)
100	CLS	−0.011	−0.001	0.026	−0.013	0.000	0.015	−0.024	−0.007	0.022
		(0.034)	(0.005)	(0.123)	(0.151)	(0.016)	(0.210)	(0.157)	(0.012)	(0.223)
	MQL	−0.011	−0.001	0.026	−0.013	−0.001	0.016	−0.022	−0.006	0.019
		(0.033)	(0.005)	(0.117)	(0.148)	(0.016)	(0.208)	(0.156)	(0.012)	(0.219)
	CML	0.006	0.005	−0.004	0.018	0.007	−0.013	0.009	0.003	−0.015
		(0.008)	(0.002)	(0.039)	(0.025)	(0.005)	(0.075)	(0.010)	(0.003)	(0.076)
300	CLS	−0.004	−0.001	0.005	−0.001	−0.002	0.008	0.000	−0.005	0.006
		(0.010)	(0.002)	(0.037)	(0.050)	(0.005)	(0.069)	(0.052)	(0.003)	(0.074)
	MQL	−0.003	−0.001	0.004	−0.001	−0.002	0.009	0.001	−0.005	0.004
		(0.010)	(0.001)	(0.034)	(0.049)	(0.005)	(0.068)	(0.051)	(0.003)	(0.073)
	CML	0.001	0.001	−0.005	0.007	0.001	−0.001	0.000	−0.001	−0.002
		(0.003)	(0.001)	(0.013)	(0.008)	(0.002)	(0.028)	(0.003)	(0.001)	(0.028)

**Table 18:** Bias and MSE for Series A of Model III $\left(\rho =\left(0.8,0.8,0.8\right)\right)$ with “burn in” samples (MSE in parentheses): CLS, MQL and CML.

*N*	Method	$\alpha_{1}^{\left(1\right)}$	$\alpha_{1}^{\left(2\right)}$	$\lambda_{1}$	$\alpha_{2}^{\left(1\right)}$	$\alpha_{2}^{\left(2\right)}$	$\lambda_{2}$	$\alpha_{3}^{\left(1\right)}$	$\alpha_{3}^{\left(2\right)}$	$\lambda_{3}$
50	CLS	−0.087	−0.040	0.214	0.018	−0.004	−0.007	0.019	0.002	−0.030
		(0.068)	(0.016)	(0.339)	(0.153)	(0.025)	(0.248)	(0.203)	(0.026)	(0.381)
	MQL	−0.011	−0.007	0.026	0.019	−0.003	−0.008	0.019	0.002	−0.031
		(0.065)	(0.014)	(0.292)	(0.155)	(0.024)	(0.244)	(0.203)	(0.026)	(0.376)
	CML	−0.016	−0.009	0.039	−0.012	−0.024	0.047	−0.109	−0.045	0.153
		(0.022)	(0.007)	(0.118)	(0.042)	(0.015)	(0.140)	(0.091)	(0.017)	(0.245)
100	CLS	−0.044	−0.017	0.103	−0.015	−0.006	0.020	−0.005	−0.003	0.008
		(0.033)	(0.008)	(0.162)	(0.075)	(0.012)	(0.132)	(0.100)	(0.013)	(0.199)
	MQL	−0.008	−0.002	0.013	−0.015	−0.006	0.020	−0.004	−0.003	0.008
		(0.030)	(0.007)	(0.137)	(0.074)	(0.012)	(0.129)	(0.099)	(0.012)	(0.197)
	CML	−0.043	−0.017	0.088	−0.057	−0.033	0.093	−0.129	−0.048	0.186
		(0.014)	(0.004)	(0.073)	(0.027)	(0.008)	(0.083)	(0.062)	(0.010)	(0.156)
300	CLS	−0.016	−0.006	0.036	0.000	−0.002	0.000	0.004	−0.001	−0.003
		(0.010)	(0.002)	(0.048)	(0.026)	(0.004)	(0.043)	(0.030)	(0.004)	(0.057)
	MQL	−0.003	−0.001	0.003	−0.001	−0.002	0.003	0.004	−0.001	−0.003
		(0.009)	(0.002)	(0.043)	(0.025)	(0.004)	(0.042)	(0.029)	(0.004)	(0.054)
	CML	−0.047	−0.020	0.097	−0.081	−0.037	0.113	−0.112	−0.046	0.169
		(0.007)	(0.002)	(0.035)	(0.016)	(0.004)	(0.038)	(0.025)	(0.004)	(0.061)

**Table 19:** Bias, bias median and MSE for Series A of Model I with “burn in“ samples.

*N*	Para.		MQL				CLS	
		Bias	Median	MSE		Bias	Median	MSE
50	$r_{1}$	−0.180	0	0.400		0.053	0	0.393
	$r_{2}$	0.390	0	1.960		0.720	0	2.894
	$r_{3}$	0.580	0	2.322		0.963	0	3.583
100	$r_{1}$	−0.099	0	0.143		−0.007	0	0.081
	$r_{2}$	0.198	0	1.142		0.491	0	1.975
	$r_{3}$	0.218	0	0.800		0.455	0	1.585
300	$r_{1}$	−0.015	0	0.015		−0.004	0	0.004
	$r_{2}$	0.018	0	0.268		0.098	0	0.416
	$r_{3}$	0.018	0	0.036		0.058	0	0.170

**Table 20:** Bias, bias median and MSE for Series A of Model II with “burn in" samples.

*N*	Para.		MQL				CLS	
		Bias	Median	MSE		Bias	Median	MSE
50	$r_{1}$	−0.071	0	0.835		0.252	0	1.394
	$r_{2}$	1.156	0	5.640		1.436	1	6.878
	$r_{3}$	1.616	1	7.974		2.046	1	10.284
100	$r_{1}$	−0.110	0	0.320		0.099	0	0.473
	$r_{2}$	1.172	0	5.662		1.477	1	7.063
	$r_{3}$	1.518	1	7.508		1.947	1	10.059
300	$r_{1}$	−0.041	0	0.055		0.027	0	0.055
	$r_{2}$	0.648	0	3.364		0.940	0	4.884
	$r_{3}$	0.574	0	3.532		0.854	0	5.338

**Table 21:** Summary statistics for the monthly counts of claimants.

	Whole Dataset	Jan.	Feb.	Mar.	Apr.	May	Jun.	Jul.	Aug.	Sep.	Oct.	Nov.	Dec.
Mean	6.1	4.2	3.8	4.6	4.9	7.0	7.1	8.5	7.5	7.2	7.2	7.2	4.4
Variance	11.8	2.2	3.3	1.8	9.0	14.7	5.9	28.9	12.5	12.0	12.2	14.8	6.9
Maximum	21	6	7	8	10	14	12	21	12	12	12	14	19
Minimum	1	2	1	3	1	2	3	3	2	2	2	2	1

**Table 22:** Threshold estimators for the monthly counts of claimants.

	Jan.	Feb.	Mar.	Apr.	May	Jun.	Jul.	Aug.	Sep.	Oct.	Nov.	Dec.
${\hat{r}}_{CLS}$	3	4	7	5	5	6	10	4	9	6	7	6
${\hat{r}}_{MQL}$	3	4	7	5	5	6	10	4	9	6	7	5

**Table 23:** The AIC and BIC of the claims data.

PSETINAR${\left(2;1,1\right)}_{12}$	AIC	BIC	PINAR${\left(1\right)}_{12}$	AIC	BIC
Pois.	586.63	596.61	Pois.	592.12	599.38
Zero-truncated Pois.	581.65	591.64	Zero-truncated Pois.	594.44	601.71
Geom.	610.45	620.43	Geom.	605.56	612.82
Zero-truncated Geom.	586.36	596.34	Zero-truncated Geom.	595.15	602.42

**Table 24:** CML estimators in the dataset.

Month	$\alpha^{\left(1\right)}$	$\alpha^{\left(2\right)}$	λ
Jan.	0.112	$8.907\times 10^{-08}$	3.819
Feb.	0.227	0.032	3.060
Mar.	0.692	-	1.969
Apr.	0.999	0.240	2.048
May	0.586	$8.521\times 10^{-09}$	4.889
Jun.	0.265	$4.316\times 10^{-08}$	5.507
Jul.	0.360	-	5.942
Aug.	0.390	-	4.186
Sep.	0.380	$3.366\times 10^{-07}$	5.218
Oct.	0.502	$1.027\times 10^{-07}$	4.044
Nov.	0.433	$2.776\times 10^{-08}$	4.990
Dec.	0.508	0.222	1.000

Remark: “-” stand for not available.

**Table 25:** PRMSE of the *h*-step-ahead point predictors.

	*h*	1	2	3	12
Conditional expectation	PSETINAR${\left(2;1,1\right)}_{12}$ (Zero-truncated Pois.)	2.641	3.019	3.433	2.929
	PINAR${\left(1\right)}_{12}$ (Zero-truncated Pois.)	2.753	3.377	3.567	3.788
	PINAR${\left(1\right)}_{12}$ (Pois.)	2.724	3.407	3.704	4.008
Conditional distribution	PSETINAR${\left(2;1,1\right)}_{12}$ (Zero-truncated Pois.)	2.814	3.000	3.109	2.930

## Data Availability

The dataset is available in the book Freeland [36].

## Appendix A

**Proof of Theorem 1.** According to Theorem 2 of Tweedie [40] (see also, Zheng and Basawa [41]), for the process defined by (2), and ∀j=1,2,⋯,T,s∈Z, we have

$$E\left(X_{j+sT}|X_{j+sT-1}=x\right)=\alpha_{j}^{\left(1\right)}xI_{j+sT-1}^{\left(1\right)}+\alpha_{j}^{\left(2\right)}xI_{j+sT-1}^{\left(2\right)}+\lambda_{j}\leq \alpha_{j,\max}x+\lambda_{j},$$

where $\alpha_{j,\max}=\max\left\{\alpha_{j}^{\left(1\right)},\alpha_{j}^{\left(2\right)}\right\}<1$. Let $K=\left[\frac{1+\lambda_{j}}{1-\alpha_{j,\max}}\right]+1$, where $\left[\cdot \right]$ denotes the integer part of a number. Then for x≥K, we have

$$E\left(X_{j+sT}|X_{j+sT-1}=x\right)\leq x-1,$$

and for x<K,

$$E\left(X_{j+sT}|X_{j+sT-1}=x\right)\leq \alpha_{j,\max}x+\lambda_{j}\leq K+\lambda_{j}<\infty .$$

Therefore, the process $\left\{X_{t}\right\}$ for t=j+sT defined in (2) is an ergodic Markov chain. □

**Proof of Proposition 2** (i) From Proposition 1, we have

$$\begin{array}{cc} Var\left(X_{j+sT}|X_{j+sT-1}\right) & =E{\left\{X_{j+sT}-E\left(X_{j+sT}|X_{j+sT-1}\right)\right\}}^{2}\\ \; & =E{\left(X_{j+sT}-\alpha_{j}^{\left(1\right)}X_{j+sT-1}I_{j+sT-1}^{\left(1\right)}-\alpha_{j}^{\left(2\right)}X_{j+sT-1}I_{j+sT-1}^{\left(2\right)}-\lambda_{j}\right)}^{2}, \end{array}$$

and

$$Var\left(X_{j+sT}|X_{j+sT-1}\right)=\sum_{k=1}^{2}\alpha_{j}^{\left(k\right)}\left(1-\alpha_{j}^{\left(k\right)}\right)X_{j+sT-1}I_{j+sT-1}^{\left(k\right)}+\sigma_{z,j}^{2},$$

with $k=1,2,j=1,\cdots ,T,s\in N_{0}$, so by substituting suitable consistent estimators of $\alpha_{j}^{\left(k\right)}$ and $\lambda_{j}$, we can get the consistent estimation of $\sigma_{z,j}^{2}$,

$$\begin{array}{cc} {\hat{\sigma }}_{1,z,j}^{2} & =\frac{1}{N}\sum_{s=0}^{N-1}{\left(X_{j+sT}-\sum_{k=1}^{2}{\hat{\alpha }}_{j}^{\left(k\right)}X_{j+sT-1}I_{j+sT-1}^{\left(k\right)}-{\hat{\lambda }}_{j}\right)}^{2}\\ \; & -\frac{1}{N}\sum_{k=1}^{2}\sum_{s=0}^{N-1}{\hat{\alpha }}_{j}^{\left(k\right)}\left(1-{\hat{\alpha }}_{j}^{\left(k\right)}\right)X_{j+sT-1}I_{j+sT-1}^{\left(k\right)}. \end{array}$$

(ii) Moreover, from model (2), we have

$$\begin{array}{cc} Var\left(X_{j+sT}\right) & =\sum_{k=1}^{2}Var\left\{\left(\alpha_{j}^{\left(k\right)}\circ X_{j+sT-1}\right)I_{j+sT-1}^{\left(k\right)}\right\}+Var\left(Z_{j+sT}\right)\\ \; & +2Cov\left\{\left(\alpha_{j}^{\left(1\right)}\circ X_{j+sT-1}\right)I_{j+sT-1}^{\left(1\right)},\left(\alpha_{j}^{\left(2\right)}\circ X_{j+sT-1}\right)I_{j+sT-1}^{\left(2\right)}\right\}, \end{array}$$

where

$$\begin{array}{cc} \; & Var\left\{\left(\alpha_{j}^{\left(k\right)}\circ X_{j+sT-1}\right)I_{j+sT-1}^{\left(k\right)}\right\}\\ = & Var\left\{E\left[\left(\alpha_{j}^{\left(k\right)}\circ X_{j+sT-1}\right)I_{j+sT-1}^{\left(k\right)}|X_{j+sT-1}\right]\right\}+E\left\{Var\left[\left(\alpha_{j}^{\left(k\right)}\circ X_{j+sT-1}\right)I_{j+sT-1}^{\left(k\right)}|X_{j+sT-1}\right]\right\}\\ = & {\alpha_{j}^{\left(k\right)}}^{2}Var\left(X_{j+sT-1}I_{j+sT-1}^{\left(k\right)}\right)+\alpha_{j}^{\left(k\right)}\left(1-\alpha_{j}^{\left(k\right)}\right)E\left(X_{j+sT-1}I_{j+sT-1}^{\left(k\right)}\right), \end{array}$$

and

$$\begin{array}{cc} \; & 2Cov\left\{\left(\alpha_{j}^{\left(1\right)}\circ X_{j+sT-1}\right)I_{j+sT-1}^{\left(1\right)},\left(\alpha_{j}^{\left(2\right)}\circ X_{j+sT-1}\right)I_{j+sT-1}^{\left(2\right)}\right\}\\ = & -2E\left\{\left(\alpha_{j}^{\left(1\right)}\circ X_{j+sT-1}\right)I_{j+sT-1}^{\left(1\right)}\right\}E\left\{\left(\alpha_{j}^{\left(2\right)}\circ X_{j+sT-1}\right)I_{j+sT-1}^{\left(2\right)}\right\}\\ = & -2\alpha_{j}^{\left(1\right)}\alpha_{j}^{\left(2\right)}E\left\{X_{j+sT-1}I_{j+sT-1}^{\left(1\right)}\right\}E\left\{X_{j+sT-1}I_{j+sT-1}^{\left(2\right)}\right\}. \end{array}$$

Note that

$$\begin{array}{cc} \; & E\left\{X_{j+sT-1}I_{j+sT-1}^{\left(1\right)}\right\}\\ = & E\left\{E\left[X_{j+sT-1}I_{j+sT-1}^{\left(1\right)}|I_{j+sT-1}^{\left(1\right)}=1\right]\right\}\\ = & E\left\{X_{j+sT-1}I_{j+sT-1}^{\left(1\right)}|I_{j+sT-1}^{\left(1\right)}=1\right\}P\left(I_{j+sT-1}^{\left(1\right)}=1\right)\\ = & E\left\{X_{j+sT-1}|X_{j+sT-1}\leq r_{j}\right\}P\left(I_{j+sT-1}^{\left(1\right)}=1\right). \end{array}$$

Let $p_{j}=P\left(I_{j+sT-1}^{\left(1\right)}=1\right)$ with $j=1,\cdots ,T,s\in N_{0}$, we can estimate it with ${\hat{p}}_{j}=\frac{1}{N}\sum_{s=0}^{N-1}I_{j+sT-1}^{\left(1\right)}$. Therefore, by substituting a suitable consistent estimator of $\alpha_{j}^{\left(k\right)}$, based on moment estimation, we can get the estimator ${\hat{\sigma }}_{2,z,j}^{2}$ in Proposition 2. □

**Proof of Theorem 2.** Let $F_{j+sT}=\sigma \left(X_{0},X_{1},\cdots ,X_{j+sT}\right)$ with $j=1,\cdots ,T,s\in N_{0}$. First, we suppose θ is known, for the following estimation equations:

$$S_{N,j}^{\left(1\right)}\left(\theta_{j},\beta_{j}\right)=\sum_{s=0}^{N-1}V_{\theta_{j}}^{-1}\left(X_{j+sT}-\alpha_{j}^{\left(1\right)}X_{j+sT-1}I_{j+sT-1}^{\left(1\right)}-\alpha_{j}^{\left(2\right)}X_{j+sT-1}I_{j+sT-1}^{\left(2\right)}-\lambda_{j}\right)X_{j+sT-1}I_{j+sT-1}^{\left(1\right)},$$

we have

$$\begin{array}{cc} \; & E\left[V_{\theta_{j}}^{-1}\left(X_{j+sT}-\alpha_{j}^{\left(1\right)}X_{j+sT-1}I_{j+sT-1}^{\left(1\right)}-\alpha_{j}^{\left(2\right)}X_{j+sT-1}I_{j+sT-1}^{\left(2\right)}-\lambda_{j}\right)X_{j+sT-1}I_{j+sT-1}^{\left(1\right)}|F_{j+sT-1}\right]\\ = & V_{\theta_{j}}^{-1}X_{j+sT-1}I_{j+sT-1}^{\left(1\right)}E\left[\left(X_{j+sT}-E\left(X_{j+sT}|X_{j+sT-1}\right)\right)|F_{j+sT-1}\right]\\ = & V_{\theta_{j}}^{-1}X_{j+sT-1}I_{j+sT-1}^{\left(1\right)}E\left[\left(X_{j+sT}-E\left(X_{j+sT}|X_{j+sT-1}\right)\right)|X_{j+sT-1}\right]\\ = & 0, \end{array}$$

and

$$\begin{array}{cc} \; & E\left[S_{s,j}^{\left(1\right)}\left(\theta_{j},\beta_{j}\right)|F_{j+sT-1}\right]\\ = & E\left[V_{\theta_{j}}^{-1}\left(X_{j+sT}\;-\;\alpha_{j}^{\left(1\right)}X_{j+sT-1}I_{j+sT-1}^{\left(1\right)}\;-\;\alpha_{j}^{\left(2\right)}X_{j+sT-1}I_{j+sT-1}^{\left(2\right)}\;-\;\lambda_{j}\right)X_{j+sT-1}I_{j+sT-1}^{\left(1\right)}+S_{s-1,j}^{\left(1\right)}\left(\theta_{j},\beta_{j}\right)|F_{j+sT-1}\right]\\ = & E\left[S_{s-1,j}^{\left(1\right)}\left(\theta_{j},\beta_{j}\right)|F_{j+sT-1}\right]\\ = & S_{s-1,j}^{\left(1\right)}\left(\theta_{j},\beta_{j}\right), \end{array}$$

so $\left\{S_{s,j}^{\left(1\right)}\left(\theta_{j},\beta_{j}\right),F_{j+sT},j=1,2,\cdots ,T,s\in N_{0}\right\}$ is a martingale. By Theorem 1.1 of Billingsley [42], we have

$$\begin{array}{cc} \; & \frac{1}{N}\sum_{s=0}^{N-1}V_{\theta_{j}}^{-2}\left(X_{j+sT}|X_{j+sT-1}\right){\left(X_{j+sT}-\alpha_{j}^{\left(1\right)}X_{j+sT-1}I_{j+sT-1}^{\left(1\right)}-\alpha_{j}^{\left(2\right)}X_{j+sT-1}I_{j+sT-1}^{\left(2\right)}-\lambda_{j}\right)}^{2}X_{j+sT-1}^{2}I_{j+sT-1}^{\left(1\right)}\\ \; & \overset{a.s.}{\to }E\left[V_{\theta_{j}}^{-2}\left(X_{j}|X_{j-1}\right){\left(X_{j}-\alpha_{j}^{\left(1\right)}X_{j-1}I_{j-1}^{\left(1\right)}-\alpha_{j}^{\left(2\right)}X_{j-1}I_{j-1}^{\left(2\right)}-\lambda_{j}\right)}^{2}X_{j-1}^{2}I_{j-1}^{\left(1\right)}\right]\\ \; & =E\{E\left[V_{\theta_{j}}^{-2}\left(X_{j}|X_{j-1}\right){\left(X_{j}-\alpha_{j}^{\left(1\right)}X_{j-1}I_{j-1}^{\left(1\right)}-\alpha_{j}^{\left(2\right)}X_{j-1}I_{j-1}^{\left(2\right)}-\lambda_{j}\right)}^{2}X_{j-1}^{2}I_{j-1}^{\left(1\right)}|X_{j-1}\right]\}\\ \; & =E\{V_{\theta_{j}}^{-2}\left(X_{j}|X_{j-1}\right)X_{j-1}^{2}I_{j-1}^{\left(1\right)}E\left[{\left(X_{j}-\alpha_{j}^{\left(1\right)}X_{j-1}I_{j-1}^{\left(1\right)}-\alpha_{j}^{\left(2\right)}X_{j-1}I_{j-1}^{\left(2\right)}-\lambda_{j}\right)}^{2}|X_{j-1}\right]\}\\ \; & =E\{V_{\theta_{j}}^{-1}\left(X_{j}|X_{j-1}\right)X_{j-1}^{2}I_{j-1}^{\left(1\right)}\}\\ \; & ≜H_{j,11}\left(\theta_{j}\right). \end{array}$$

Thus, by the central limit theorem of martingale, we get

$$\frac{1}{\sqrt{N}}S_{N,j}^{\left(1\right)}\left(\theta_{j},\beta_{j}\right)\overset{L}{\to }N\left(0,H_{j,11}\left(\theta_{j}\right)\right).$$

Similarly,

$$\frac{1}{\sqrt{N}}S_{N,j}^{\left(2\right)}\left(\theta_{j},\beta_{j}\right)\overset{L}{\to }N\left(0,H_{j,22}\left(\theta_{j}\right)\right),$$

$$\frac{1}{\sqrt{N}}S_{N,j}^{\left(3\right)}\left(\theta_{j},\beta_{j}\right)\overset{L}{\to }N\left(0,H_{j,33}\left(\theta_{j}\right)\right),$$

where

$$S_{N,j}^{\left(2\right)}\left(\theta_{j},\beta_{j}\right)=\sum_{s=0}^{N-1}V_{\theta_{j}}^{-1}\left(X_{j+sT}-\alpha_{j}^{\left(1\right)}X_{j+sT-1}I_{j+sT-1}^{\left(1\right)}-\alpha_{j}^{\left(2\right)}X_{j+sT-1}I_{j+sT-1}^{\left(2\right)}-\lambda_{j}\right)X_{j+sT-1}I_{j+sT-1}^{\left(2\right)},$$

and

$$S_{N,j}^{\left(3\right)}\left(\theta_{j},\beta_{j}\right)=\sum_{s=0}^{N-1}V_{\theta_{j}}^{-1}\left(X_{j+sT}-\alpha_{j}^{\left(1\right)}X_{j+sT-1}I_{j+sT-1}^{\left(1\right)}-\alpha_{j}^{\left(2\right)}X_{j+sT-1}I_{j+sT-1}^{\left(2\right)}-\lambda_{j}\right).$$

For any $c={\left(c_{1},c_{2},\cdots ,c_{T}\right)}^{\prime }\in R^{3T}\setminus \left(0,0,\cdots ,0\right)$, $c_{j}={\left(c_{j}^{\left(1\right)},c_{j}^{\left(2\right)},c_{j}^{\left(3\right)}\right)}^{\prime }$ with j=1,2,⋯,T, to simplify, let

$$j+sT\neq i+kT,i,j=1,2,\cdots ,T,s,k\in N_{0},$$

$$u_{j,s}=c_{j}^{\left(1\right)}X_{j+sT-1}I_{j+sT-1}^{\left(1\right)}+c_{j}^{\left(2\right)}X_{j+sT-1}I_{j+sT-1}^{\left(2\right)}+c_{j}^{\left(3\right)},$$

$$w_{j,s}=X_{j+sT}-E\left(X_{j+sT}|X_{j+sT-1}\right)=X_{j+sT}-\alpha_{j}^{\left(1\right)}X_{j+sT-1}I_{j+sT-1}^{\left(1\right)}-\alpha_{j}^{\left(2\right)}X_{j+sT-1}I_{j+sT-1}^{\left(2\right)}-\lambda_{j},$$

and $n\left(T,N\right)$ is a constant associated with *N* and *T*, then

(A1) $$\begin{array}{cc} \; & E{\left[\sum_{j=1}^{T}\sum_{s=0}^{N-1}V_{\theta_{j}}^{-1}\left(X_{j+sT}-E\left(X_{j+sT}|X_{j+sT-1}\right)\right)\left(c_{j}^{\left(1\right)}X_{j+sT-1}I_{j+sT-1}^{\left(1\right)}+c_{j}^{\left(2\right)}X_{j+sT-1}I_{j+sT-1}^{\left(2\right)}+c_{j}^{\left(3\right)}\right)\right]}^{2}\\ \; & =\sum_{j=1}^{T}\sum_{s=0}^{N-1}E\left[V_{\theta_{j}}^{-2}w_{j,s}^{2}u_{j,s}^{2}\right]+n\left(T,N\right)E\left[V_{\theta_{j}}^{-1}V_{\theta_{i}}^{-1}w_{j,s}w_{i,k}u_{j,s}u_{i,k}\right], \end{array}$$

for the first item in the right side of Equation (A1), we have

$$\begin{array}{cc} \; & E\left[V_{\theta_{j}}^{-2}w_{j,s}^{2}u_{j,s}^{2}\right]\\ = & E\{E\left[V_{\theta_{j}}^{-2}w_{j,s}^{2}u_{j,s}^{2}|X_{j+sT-1}\right]\}\\ = & E\{V_{\theta_{j}}^{-2}u_{j,s}^{2}E\left[w_{j,s}^{2}|X_{j+sT-1}\right]\}\\ = & E\left[V_{\theta_{j}}^{-1}{\left(c_{j}^{\left(1\right)}X_{j+sT-1}I_{j+sT-1}^{\left(1\right)}+c_{j}^{\left(2\right)}X_{j+sT-1}I_{j+sT-1}^{\left(2\right)}+c_{j}^{\left(3\right)}\right)}^{2}\right], \end{array}$$

for the second item in the right side of Equation (A1), we have

$$\begin{array}{cc} \; & E\left[V_{\theta_{j}}^{-1}V_{\theta_{i}}^{-1}w_{j,s}w_{i,k}u_{j,s}u_{i,k}\right]\\ = & E\{V_{\theta_{j}}^{-1}V_{\theta_{i}}^{-1}u_{j,s}u_{i,k}E\left[w_{j,s}w_{i,k}|X_{j+sT-1},X_{i+kT-1}\right]\}\\ = & 0, \end{array}$$

which imply that $Cov\left(S_{N,j},S_{N,i}\right)=0$, where $S_{N,j}={\left(S_{N,j}^{\left(1\right)}\left(\theta_{j},\beta_{j}\right),S_{N,j}^{\left(2\right)}\left(\theta_{j},\beta_{j}\right),S_{N,j}^{\left(3\right)}\left(\theta_{j},\beta_{j}\right)\right)}^{\prime },$∀i,j=1,2,⋯,T,i≠j, then we have

$$\begin{array}{cc} \; & \frac{c_{j}^{T}}{\sqrt{N}}{\left(S_{N,j}^{\left(1\right)}\left(\theta_{j},\beta_{j}\right),S_{N,j}^{\left(2\right)}\left(\theta_{j},\beta_{j}\right),S_{N,j}^{\left(3\right)}\left(\theta_{j},\beta_{j}\right)\right)}^{\prime }\\ \; & =\frac{1}{\sqrt{N}}\sum_{i=1}^{3}c_{j}^{\left(i\right)}S_{N,j}^{\left(i\right)}\left(\theta_{j},\beta_{j}\right)\\ \; & =\frac{1}{\sqrt{N}}\sum_{s=0}^{N-1}V_{\theta_{j}}^{-1}\left[X_{j+sT}-E\left(X_{j+sT}|X_{j+sT-1}\right)\right]\left(c_{j}^{\left(1\right)}X_{j+sT-1}I_{j+sT-1}^{\left(1\right)}+c_{j}^{\left(2\right)}X_{j+sT-1}I_{j+sT-1}^{\left(2\right)}+c_{j}^{\left(3\right)}\right)\\ \; & \overset{L}{\to }N\left(0,E\left[V_{\theta_{j}}^{-1}\left(X_{j}|X_{j-1}\right){\left(c_{j}^{\left(1\right)}X_{j-1}I_{j-1}^{\left(1\right)}+c_{j}^{\left(2\right)}X_{j-1}I_{j-1}^{\left(2\right)}+c_{j}^{\left(3\right)}\right)}^{2}\right]\right),as\;N\to \infty , \end{array}$$

therefore, the $\frac{c_{j}^{T}}{\sqrt{N}}{\left(S_{N,j}^{\left(1\right)}\left(\theta_{j},\beta_{j}\right),S_{N,j}^{\left(2\right)}\left(\theta_{j},\beta_{j}\right),S_{N,j}^{\left(3\right)}\left(\theta_{j},\beta_{j}\right)\right)}^{\prime }$ converges to a normal distribution with mean zero and variance $E\left[V_{\theta_{j}}^{-1}\left(X_{j}|X_{j-1}\right){\left(c_{j}^{\left(1\right)}X_{j-1}I_{j-1}^{\left(1\right)}+c_{j}^{\left(2\right)}X_{j-1}I_{j-1}^{\left(2\right)}+c_{j}^{\left(3\right)}\right)}^{2}\right]$. Thus, by Cramer-wold device, it follows that

$$\begin{array}{cc} \; & \frac{1}{\sqrt{N}}\left(\begin{array}{c} S_{N,1}^{\left(1\right)}\left(\theta_{1},\beta_{1}\right)\\ S_{N,1}^{\left(2\right)}\left(\theta_{1},\beta_{1}\right)\\ S_{N,1}^{\left(3\right)}\left(\theta_{1},\beta_{1}\right)\\ \vdots \\ S_{N,T}^{\left(1\right)}\left(\theta_{T},\beta_{T}\right)\\ S_{N,T}^{\left(2\right)}\left(\theta_{T},\beta_{T}\right)\\ S_{N,T}^{\left(3\right)}\left(\theta_{T},\beta_{T}\right) \end{array}\right)\overset{L}{\to }N\left(0,\left[\begin{array}{cccc} H_{1}\left(\theta \right) & 0 & \cdots & 0\\ 0 & H_{2}\left(\theta \right) & \cdots & 0\\ \vdots & \vdots & \ddots & \vdots \\ 0 & 0 & \cdots & H_{T}\left(\theta \right) \end{array}\right]\right), \end{array}$$

the 0’s are $\left(3\times 3\right)$-null matrices. Now, we replace $V_{\theta_{j}}\left(X_{j+sT}|X_{j+sT-1}\right)$ by $V_{{\hat{\theta }}_{j}}\left(X_{j+sT}|X_{j+sT-1}\right)$, where ${\hat{\theta }}_{j}$ is a consistent estimator of $\theta_{j}$. We aim to get the result

(A2) $$\begin{array}{cc} \; & \frac{1}{\sqrt{N}}\left(\begin{array}{c} S_{N,1}^{\left(1\right)}\left({\hat{\theta }}_{1},\beta_{1}\right)\\ S_{N,1}^{\left(2\right)}\left({\hat{\theta }}_{1},\beta_{1}\right)\\ S_{N,1}^{\left(3\right)}\left({\hat{\theta }}_{1},\beta_{1}\right)\\ \vdots \\ S_{N,T}^{\left(1\right)}\left({\hat{\theta }}_{T},\beta_{T}\right)\\ S_{N,T}^{\left(2\right)}\left({\hat{\theta }}_{T},\beta_{T}\right)\\ S_{N,T}^{\left(3\right)}\left({\hat{\theta }}_{T},\beta_{T}\right) \end{array}\right)\overset{L}{\to }N\left(0,\left[\begin{array}{cccc} H_{1}\left(\theta \right) & 0 & \cdots & 0\\ 0 & H_{2}\left(\theta \right) & \cdots & 0\\ \vdots & \vdots & \ddots & \vdots \\ 0 & 0 & \cdots & H_{T}\left(\theta \right) \end{array}\right]\right). \end{array}$$

To prove (A2), we need to check the following conclusion

(A3) $$\frac{1}{\sqrt{N}}S_{N,j}^{\left(i\right)}\left({\hat{\theta }}_{j},\beta_{j}\right)-\frac{1}{\sqrt{N}}S_{N,j}^{\left(i\right)}\left(\theta_{j},\beta_{j}\right)\overset{P}{\to }0,j=1,2,\cdots ,T,i=1,2,3,N\to \infty .$$

For ∀ϵ>0, ∃δ>0, we have

$$\begin{array}{cc} P\left(|\frac{1}{\sqrt{N}}S_{N}^{\left(1\right)}\left({\hat{\theta }}_{j},\beta_{j}\right)-\frac{1}{\sqrt{N}}S_{N}^{\left(1\right)}\left(\theta_{j},\beta_{j}\right)|>\epsilon \right)\leq & \sum_{k=1}^{2}P\left(|\theta_{j_{1}}^{\left(k\right)}-\theta_{j}^{\left(k\right)}|>\delta \right)+P\left(|\sigma_{z,j_{1}}^{2}-\sigma_{z,j}^{2}|>\delta \right)\\ \; & +P\left(\underset{D}{\sup}\left|\frac{1}{\sqrt{N}}S_{N}^{\left(1\right)}\left(\theta_{j_{1}},\beta_{j}\right)-\frac{1}{\sqrt{N}}S_{N}^{\left(1\right)}\left(\theta_{j},\beta_{j}\right)\right|>\epsilon \right), \end{array}$$

where $\theta_{j_{1}}={\left(\theta_{j_{1}}^{\left(1\right)},\theta_{j_{1}}^{\left(2\right)},\sigma_{z,j_{1}}^{2}\right)}^{\prime }$, $D=\{\theta_{j_{1}}:|\theta_{j_{1}}^{\left(1\right)}-\theta_{j}^{\left(1\right)}|<\delta ,|\theta_{j_{1}}^{\left(2\right)}-\theta_{j}^{\left(2\right)}|<\delta ,\sigma_{z,j_{1}}^{2}-\sigma_{z,j}^{2}|<\delta \}$. If ${\hat{\theta }}_{j}$ is a consistent estimator of $\theta_{j}$, then we just need to prove that

$$P\left(\underset{D}{\sup}\left|\frac{1}{\sqrt{N}}S_{N}^{\left(1\right)}\left(\theta_{j_{1}},\beta_{j}\right)-\frac{1}{\sqrt{N}}S_{N}^{\left(1\right)}\left(\theta_{j},\beta_{j}\right)\right|>\epsilon \right)\overset{P}{\to }0,N\to \infty .$$

By the Markov inequality,

$$\begin{array}{cc} \; & P\left(\underset{D}{\sup}\left|\frac{1}{\sqrt{N}}S_{N}^{\left(1\right)}\left(\theta_{j_{1}},\beta_{j}\right)-\frac{1}{\sqrt{N}}S_{N}^{\left(1\right)}\left(\theta_{j},\beta_{j}\right)\right|>\epsilon \right)\\ \; & \leq \frac{1}{\epsilon^{2}}E\left(\underset{D}{\sup}{\left(\frac{1}{\sqrt{N}}S_{N}^{\left(1\right)}\left(\theta_{j_{1}},\beta_{j}\right)-\frac{1}{\sqrt{N}}S_{N}^{\left(1\right)}\left(\theta_{j},\beta \right)\right)}^{2}\right)\\ \; & \leq \frac{1}{\epsilon^{2}}E\left\{\underset{D}{\sup}\frac{1}{N}\left[\sum_{s=0}^{N-1}{\left(V_{\theta_{j_{1}}}^{-1}-V_{\theta_{j}}^{-1}\right)}^{2}{\left(X_{j+sT}-E\left(X_{j+sT}|X_{j+sT-1}\right)\right)}^{2}X_{j+sT-1}^{2}I_{j+sT-1}^{\left(1\right)}\right]\right\}\\ \; & =\frac{1}{\epsilon^{2}}E\left[\underset{D}{\sup}{\left(V_{\theta_{j_{1}}}^{-1}\left(X_{j}|X_{j-1}\right)-V_{\theta_{j}}^{-1}\left(X_{j}|X_{j-1}\right)\right)}^{2}{\left(X_{j}-\alpha_{j}^{\left(1\right)}X_{j-1}I_{j-1}^{\left(1\right)}-\alpha_{j}^{\left(2\right)}X_{j-1}I_{j-1}^{\left(2\right)}-\lambda_{j}\right)}^{2}X_{j-1}^{2}I_{j-1}^{\left(1\right)}\right]\\ \; & =\frac{1}{\epsilon^{2}}E\left\{\underset{D}{\sup}\frac{{\left[\left(\theta_{j}^{\left(1\right)}-\theta_{j_{1}}^{\left(1\right)}\right)X_{j-1}I_{j-1}^{\left(1\right)}+\left(\theta_{j}^{\left(1\right)}-\theta_{j_{1}}^{\left(2\right)}\right)X_{j-1}I_{j-1}^{\left(2\right)}+\left(\sigma_{z,j}^{2}-\sigma_{z,j_{1}}^{2}\right)\right]}^{2}}{V_{\theta_{j_{1}}}^{2}\left(X_{j}|X_{j-1}\right)V_{\theta_{j}}\left(X_{j}|X_{j-1}\right)}X_{j-1}^{2}I_{j-1}^{\left(1\right)}\right\}\\ \; & \leq \frac{1}{\epsilon^{2}}\underset{D}{\sup}\{[{\left(\theta_{j}^{\left(1\right)}-\theta_{j_{1}}^{\left(1\right)}\right)}^{2}m_{1}+{\left(\theta_{j}^{\left(2\right)}-\theta_{j_{1}}^{\left(2\right)}\right)}^{2}m_{2}+{\left(\sigma_{z,j}^{2}-\sigma_{z,j_{1}}^{2}\right)}^{2}m_{3}+2m_{4}|\theta_{j}^{\left(1\right)}-\theta_{j_{1}}^{\left(1\right)}\left|\right|\theta_{j}^{\left(2\right)}-\theta_{j_{1}}^{\left(2\right)}|\\ \; & +2m_{5}|\theta_{j}^{\left(1\right)}-\theta_{j_{1}}^{\left(1\right)}\left|\right|\sigma_{z,j}^{2}-\sigma_{z,j_{1}}^{2}|+2m_{6}|\theta_{j}^{\left(2\right)}-\theta_{j_{1}}^{\left(2\right)}\left|\right|\sigma_{z,j}^{2}-\sigma_{z,j_{1}}^{2}\left|\right]X_{j-1}^{2}I_{j-1}^{\left(1\right)}\}\\ \; & \leq \frac{c\delta^{2}}{\epsilon^{2}}, \end{array}$$

where $m_{1},m_{2},\cdots ,m_{6}$ are some finite moments of process $\left\{X_{t}\right\}$ under assumption (C2), and *c* is a positive constant. A similar argument can be used for $\frac{1}{\sqrt{N}}S_{N,j}^{\left(2\right)}\left(\theta_{j},\beta_{j}\right)$ and $\frac{1}{\sqrt{N}}S_{N,j}^{\left(3\right)}\left(\theta_{j},\beta_{j}\right)$, j=1,⋯,T. Let δ→0, we can get (A3). By the ergodic theorem, we have

$$\frac{1}{N}Q_{N}\overset{P}{\to }H\left(\theta \right).$$

After some calculation, we have

$$\begin{array}{cc} \; & Q_{N}\left({\hat{\beta }}_{MQL}-\beta \right)\\ = & {\left(S_{N,1}^{\left(1\right)}\left({\hat{\theta }}_{1},\beta_{1}\right),S_{N,1}^{\left(2\right)}\left({\hat{\theta }}_{1},\beta_{1}\right),S_{N,1}^{\left(3\right)}\left({\hat{\theta }}_{1},\beta_{1}\right),\cdots ,S_{N,T}^{\left(1\right)}\left({\hat{\theta }}_{T},\beta_{T}\right),S_{N,T}^{\left(2\right)}\left({\hat{\theta }}_{T},\beta_{T}\right),S_{N,T}^{\left(3\right)}\left({\hat{\theta }}_{T},\beta_{T}\right)\right)}^{\prime }, \end{array}$$

Therefore,

$$\sqrt{N}\left({\hat{\beta }}_{MQL}-\beta \right)\overset{L}{\to }N\left(0,H^{-1}\left(\theta \right)\right).$$

This completes the proof. □
